# Effects of Three Anthelmintics on the Transcriptome of FHs 74 Int Enterocytes

**DOI:** 10.3390/tropicalmed11090257

**Published:** 2026-09-10

**Authors:** Kornkanok Nakudom, Amornrat Geadkaew-Krenc, Wansika Phadungsil, Rudi Grams

**Affiliations:** Graduate Program in Biomedical Sciences, Faculty of Allied Health Sciences, Thammasat University, Khlong Luang, Pathum Thani 12120, Thailand; snuk.k@hotmail.com (K.N.); gamornra@tu.ac.th (A.G.-K.); wansika_tu@hotmail.com (W.P.)

**Keywords:** anthelmintics, albendazole, ivermectin, praziquantel, human intestinal cells, transcriptomics

## Abstract

Helminth infections continue to present a substantial public health challenge worldwide, particularly in tropical and developing areas. Despite the widespread use of anthelmintic drugs for treatment and control, their effects on host cells remain poorly understood. In this study, the three commonly used anthelmintics albendazole, ivermectin, and praziquantel were investigated by transcriptome analysis for their effects on gene activity in the human intestinal cell line FHs 74 Int after 24-h exposure. The drug concentrations used were substantially higher than in the treatment of helminthiases but did not affect cell viability after 24 h and less than 50% after 72 h. Praziquantel had negligible toxic effects on the intestinal cells at the highest achievable concentration of 1 mM whereas albendazole and ivermectin were already effective at low micromolar concentrations and were tested at 1 µM and 15 µM concentrations. Consistent with the observed cytotoxicity, transcriptome analysis showed no transcriptional changes for 1 mM praziquantel at low cutoff criteria of adjusted *p*-value (padj) ≤ 0.05, log_2_ fold change ≥ 0. At the same padj but with log_2_ fold change ≥ 2, 17 upregulated and 2 downregulated genes were found after treatment with 1 µM albendazole and 106 upregulated and 65 downregulated genes with 15 µM ivermectin. Analysis of these genes showed that albendazole caused a modest upregulation of cell cycle related processes, especially mitotic spindle formation, and chromosome segregation. The cells showed a stronger response to ivermectin and the data suggested inhibition of cell proliferation through downregulated transcription factors and nuclear receptors and stimulation of cell growth and cell survival mechanisms through upregulated metabolic enzymes.

## 1. Introduction

Infestation with parasitic worms is a significant public health problem that affects more than one billion people worldwide, especially in low-income and developing regions [1,2]. Chronic infection can lead to malnutrition, anemia, impaired physical and cognitive development, and reduced productivity [3,4]. Anthelmintic drug intervention and mass drug administration programs are used to control these diseases. However, many of the common anthelmintics have side effects with well-described symptoms. It is important to understand not only their antiparasitic efficacy but also their potential effects on the host at the cellular level.

Albendazole (ABZ), ivermectin (IVM) and praziquantel (PZQ) (PubChem Compound CID: 2082, 6321424, 4891, National Center for Biotechnology Information) are widely effective agents used to treat and control helminth infections. These drugs are structurally unrelated and have different pharmacology and pharmacokinetics. Albendazole is a broad-spectrum anthelmintic drug for the treatment of cestodes but may also be used for trematode and roundworm infections. It belongs to the benzimidazole family, binds selectively to β-tubulin in parasite cells and inhibits polymerization into microtubules and interrupts glucose transport. This makes the parasites unable to generate sufficient energy for metabolic processes, leads to depletion of glycogen stores and results in parasite immobilization and death [5,6,7]. The recommended dosage of albendazole in human infection is 400 mg.

Ivermectin is well known for its application in roundworm infections such as onchocerciasis, strongyloidiasis and filariasis. It is a semi-synthetic antiparasitic agent derived from avermectins that were isolated from the fermentation products of *Streptomyces avermitilis*. It causes parasite paralysis by selective and high-affinity binding to glutamate-gated ion channels of invertebrate nerve and muscle cells, which then leads to increased chloride ion influx and results in hyperpolarization of the cell membrane [8,9,10]. In humans, the recommended dosage of ivermectin depends on the kind of infection including a single oral dose of 150 μg/kg (onchocerciasis), subcutaneous injection of 200 μg/kg/day for two days (strongyloidiasis) and oral ivermectin at 200 μg/kg (lymphatic filariasis).

Praziquantel is widely used in trematode infections, e.g., opisthorchiasis, schistosomiasis but can also be applied for other helminths including cestodes. It is a derivative of pyrazino-isoquinoline from the thioxantonic group that increases the permeability of parasite cell membranes to calcium ions, resulting in calcium influx to parasite muscle cells, causing chronic muscle contraction, paralysis, and parasite death [11,12,13]. In humans, the recommended dosage of praziquantel for fluke infection is a single oral dose of 40 mg/kg. Ivermectin has the longest half-life at 18 h, followed by albendazole at 8–12 h and the short-lived praziquantel at 0.8–1.5 h. However, general side effects of all three drugs were observed including headaches, nausea, vomiting, dizziness and stomach pain as listed in NIH NLM MedlinePlus (https://medlineplus.gov/ accessed on 29 June 2026) under accessions a610019 (ABZ), a607069 (IVM), a608048 (PZQ).

Transcriptomic technologies such as RNA sequencing have been widely used to study the gene expression changes in different conditions [14]. Conventional pharmacological studies have focused on the direct effects of drugs on helminths, whereas recent studies have shown that anthelmintic compounds can also modulate host cellular processes, including gene expression, immune responses, and metabolic regulation [15,16,17]. A transcriptomic examination of drug-induced changes in global gene expression profiles is a powerful approach for exploring differential gene expression and gaining greater insight into mechanisms of drug effects, off-target responses, and interactions between the drug and the host.

In this study, we have compared the transcriptomic responses of FHs 74 Int enterocytes after exposure to high concentrations of albendazole, ivermectin and praziquantel. Differential gene expression, functional enrichment analysis and protein-protein interaction network were performed for each drug to provide comprehensive gene expression data. The data do not concern the routine use of these drugs at much lower dosage in helminthiases but advances our understanding on their toxic potential at the cellular and molecular level.

## 2. Materials and Methods

### 2.1. Cell Culture and Drug Treatment

Human fetal small intestine FHs 74 Int cells (JCRB9076) were obtained from JCRB Cell Bank (Japanese Collection of Research Bioresources Cell Bank, Osaka, Japan) at passage 27. The source of JCRB9076, as stated by JCRB, was ATCC (American Type Culture Collection) CCL-241 (https://www.atcc.org/api/pdf/product-sheet?id=CCL-241 (accessed on 29 June 2026)). The cells were maintained in Dulbecco’s modified Eagle’s medium (Corning, Manassas, VA, USA) supplemented with 30 ng/mL epidermal growth factor (EGF; Sigma-Aldrich, St. Louis, MO, USA), 10% heat-inactivated fetal bovine serum (Gibco, Grand Island, NY, USA), and 100 U/mL antibiotic-antimycotic (Gibco, Grand Island, NY, USA) at 37 °C with 5% CO_2_ in a humidified incubator.

Albendazole (ABZ, A4673), ivermectin (IVM, I8898), and praziquantel (PZQ, P4668) were obtained from Sigma-Aldrich (Sigma, St. Louis, MO, USA). ABZ, IVM and PZQ were dissolved in DMSO at concentrations of 100, 50 and 200 mM, respectively. The stock drug solutions were diluted with culture medium and DMSO to 2× final concentrations containing 1% DMSO.

The cytotoxicity of albendazole, ivermectin, and praziquantel was determined by MTT assay with three independent biological replicates with cells at passage 32, 33, and 34. Three technical replicates (wells) were made from each biological replicate. Cells were seeded into 96-well plates at a density of 1 × 10^4^ cells in 100 µL medium per well. After 24 h, cells were treated with different concentrations of albendazole (0.01–500 µM), ivermectin (10–25 µM) and praziquantel (300–1000 µM) by adding 100 µL of 2× concentrated drugs for 24, 48 and 72 h. Then, 20 µL of 5 mg/mL MTT solution (Invitrogen^TM^, Thermo Fisher Scientific, Carlsbad, CA, USA) was added and incubated at 37 °C for 3 h in a dark place. After that, the medium was removed, and 100 µL DMSO was added to each well to dissolve the formazan crystals. The absorbance was measured at 540 nm using a Varioskan Flash spectral scanning multimode reader (Thermo Fisher Scientific, Carlsbad, CA, USA). The recorded data were analyzed and visualized in Prism version 10.4.2, GraphPad Software, LLC (Boston, MA, USA).

### 2.2. Isolation of RNA from Treated/Untreated Cells, Library Preparation and Sequencing

The highest non-toxic concentration at 24 h was used for each drug. FHs 74 Int cells at passage 33 were seeded at a density of 5 × 10^5^ cells/well in 6-well plates and treated with albendazole (1 µM), ivermectin (15 µM), praziquantel (1000 µM) or 0.5% DMSO (control) for 24 h. Total RNA was extracted from the monolayer cells of three independent biological samples without technical replicates for each drug using a RNeasy^®^ Plus Mini Kit (QIAgen, Hilden, Germany). RNA concentration and purity were determined by measuring the optical density at A260, A280, and A230 using a NanoDrop™ One Microvolume UV-Vis Spectrophotometer (Thermo Scientific, Waltham, MA, USA). RNA integrity and genomic DNA contamination was detected by a Bioanalyzer RNA 6000 Pico assay (Agilent Technologies, Santa Clara, CA, USA). All RNA samples were shipped to Novogene for RNA sequencing.

The paired-end cDNA libraries were prepared using Fast RNA-seq Lib Prep Kit V2 (ABclonal Technology, Woburn, MA, USA) and sequenced on an Illumina^®^ Novaseq™ 6000 150 PE platform (Illumina^®^, San Diego, CA, USA). FastQ [18] was used for quality control of the raw sequencing reads. High-quality clean reads were obtained by removing reads containing adapters, reads containing poly-N and low-quality reads from the raw data. The parameters of quality control relied on per-base quality Phred Q scores 20 and 30 (Q20 and Q30), sequencing error rate distribution, and GC-content. High-quality clean reads were aligned to the human reference genome GRCh38 using HISAT2 v2.0.5 [19].

The number of uniquely mapped reads on each transcript were normalized and expressed as Fragments Per Kilobase of transcript per Million mapped reads (FPKM) using featureCounts v1.5.0-p3 [20]. The FPKM-normalized values were transformed into log_2_(FPKM+1) values and used in gene expression analysis. The similarity and viability of transcriptomic data across biological replicates were examined using kernel density estimation (KDE) curves, violin plots, boxplots and Pearson’s correlation coefficients (R^2^) to ensure the reliability of subsequent differential expression analysis.

Differential gene expression analysis was performed using the DESeq2 R package v1.20.0 [21] to evaluate changes in gene expression associated with drug treatment. DESeq2 analysis provides a statistical value in the magnitude of change (log_2_ fold change) and the magnitude of difference (*p*-value) using a model based on the negative binomial distribution. The magnitude of change was used to determine for upregulation and downregulation. In this part, upregulated differentially expressed genes (DEGs) were considered at log_2_ fold change ≥ 0 and downregulated DEGs were considered at log_2_ fold change ≤ 0.

Functional annotation of DEGs was performed using Gene Ontology (GO) [22] enrichment analyses with PAN-GO [23] at https://functionome.geneontology.org/ (accessed on 28 July 2026). GO enrichment analysis classified DEGs into biological process, molecular function, and cellular component categories. Enriched GO terms with an adjusted *p*-value < 0.05 were considered statistically significant.

Protein-protein interaction (PPI) network analysis including functional enrichment analysis was performed using the STRING database [24] through the stringApp v2.2.0 and MCODE v2.0.3 [25] in Cytoscape v3.10.4 [26]. Stronger interactions of proteins are represented by thicker lines in the graphical output of Cytoscape.

## 3. Results

### 3.1. Cytotoxic Effects of Albendazole, Ivermectin and Praziquantel on FHs 74 Int Cells

**Figure 1:**
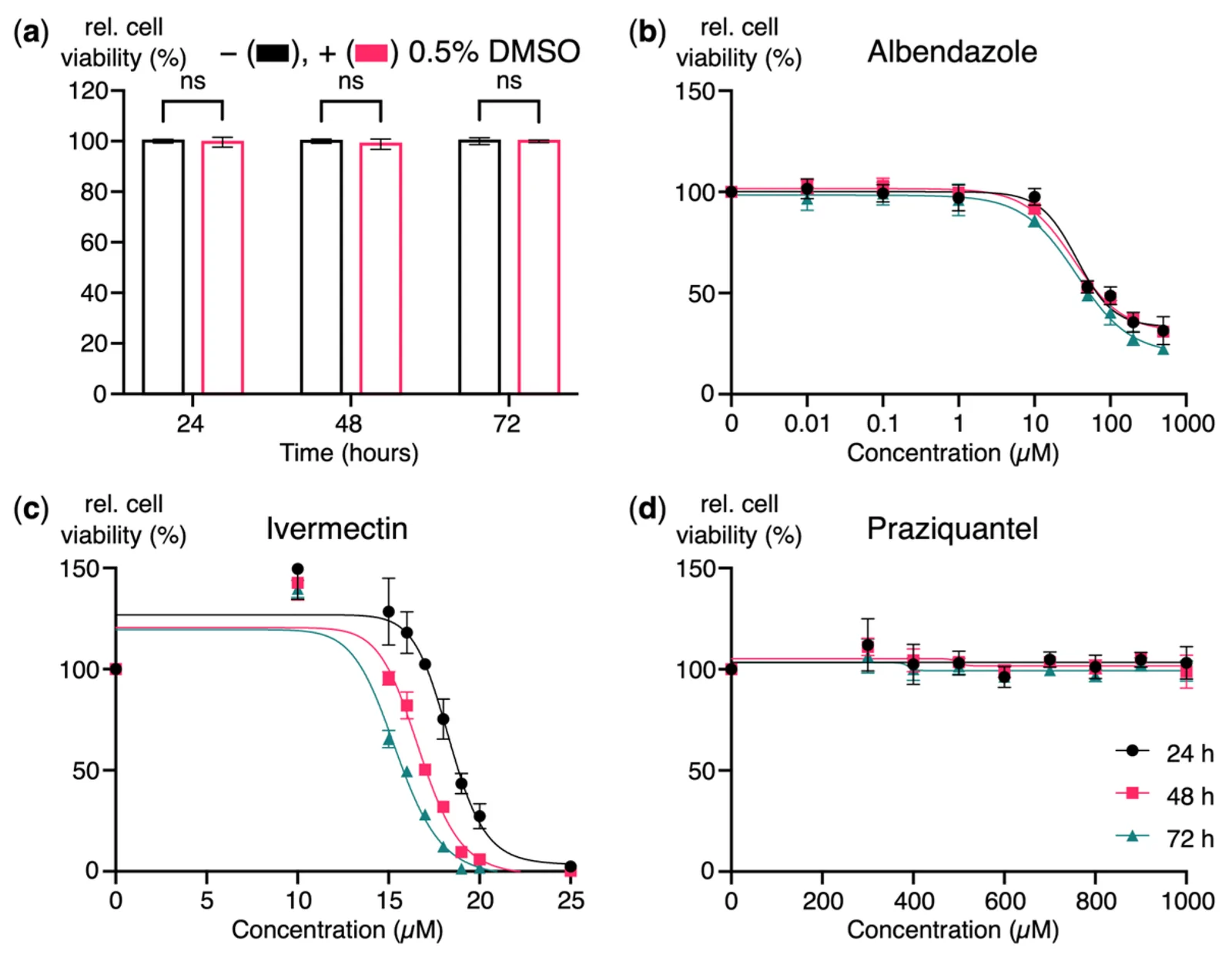
(**a**) Viability of FHs 74 Int cells cultured without and with 0.5% DMSO determined by MTT assay after 24, 48, 72 h, ns indicates non-significant difference as tested by unpaired *t*-test. Cytotoxicity of (**b**) albendazole, (**c**) ivermectin and (**d**) praziquantel on FHs 74 Int cells determined by MTT assay after 24 h (black), 48 h (red), and 72 h (green). Nonlinear fits of the data are shown with the data points as mean ± SD of per cent cell viability.

In this study, the non-malignant FHs 74 Int cell line was used. It has been described as an enterocyte with epithelial morphology that can be used to study normal gastrointestinal functions. The cytotoxic effects of various concentrations of albendazole, ivermectin, and praziquantel on these cells were evaluated following 24, 48, and 72 h of treatment. Cell viability was expressed as a percentage relative to the control (0.5% DMSO), which was set as 100%. Cell viability of the control at 24, 48, and 72 h was not significantly different from viability of cells grown without DMSO as tested by unpaired *t*-test (*p* < 0.05, Figure 1a). The three drugs showed distinct results as might be expected from their different chemical nature and mode of action.

Albendazole showed minimal or no cytotoxic effects on the cells at low concentrations (≤1 µM) at all time points. At higher concentrations (≥10 µM), cell viability decreased with increasing concentration and incubation time. Marked cytotoxicity was observed at concentrations ≥100 µM, particularly after 72 h of exposure but never reached 100% (Figure 1b).

Ivermectin also induced concentration- and time-dependent cytotoxic effects. Treatment at 10 µM ivermectin resulted in increased cell survival compared to the untreated control. Concentrations ≥15 µM quickly reduced cell viability in correlation with time. At 20–25 µM, complete cytotoxicity was observed depending on incubation time (Figure 1c).

Praziquantel did not cause cytotoxicity at all tested concentrations and time points. Cell viability remained comparable to the DMSO control group even at a 1000 µM concentration. This was the highest concentration achievable at 0.5% DMSO (Figure 1d).

Based on these results, the highest concentrations of each drug that showed minimal or no cytotoxic effects after 24 h were selected for subsequent experiments. These concentrations were 1 µM albendazole, 15 µM ivermectin, and 1000 µM praziquantel.

### 3.2. Isolation of RNA from FHs 74 Int Cells and Transcriptome Sequencing

Three independent RNA samples from cells in separate wells were prepared for each condition and analyzed for concentration, quality, and integrity. The summary of the RNA quality assessment is shown in Table 1. The RNA concentrations of the samples ranged from 11–20 ng/µL, and the total amount was greater than 0.4 µg for each sample. Electropherograms of all RNA samples showed two smooth-shaped peaks of 18S and 28S rRNA. Additionally, the RNA integrity number (RIN) for all RNA samples was greater than 6.9, indicating high RNA quality. All RNA samples were found to be of high quality and were used for RNA sequencing.

Paired-end cDNA libraries were prepared and sequenced on an Illumina^®^ NovaSeq™ 6000 150 PE platform. High numbers of raw reads were generated, ranging from 4.08 × 10^7^ to 6.68 × 10^7^ reads per sample. After quality control filtering, 97.69–98.94% of the raw reads were retained (4.04 × 10^7^ to 6.58 × 10^7^ reads per sample), indicating minimal loss during the filtering process and overall high sequencing quality. Following quality filtering, the reads were aligned to the human GRCh38 reference genome and the alignment statistics are summarized in Table 2. Across all samples, a high proportion of clean reads was successfully aligned to the reference genome, with total mapping rates ranging from 95.81% to 97.11%, indicating consistent and reliable alignment performance. The sequence data of the twelve samples are available at the Sequence Read Archive (SRA) under BioProject accession PRJNA1483491.

Classification of aligned reads showed that the majority were mapped to a single genomic location. Uniquely mapped reads accounted for 93.41–95.04% of the total clean reads, whereas reads mapped to multiple genomic locations represented only a small fraction of the dataset (2.06–2.41%). The high proportion of uniquely aligned reads supports the suitability of the data for accurate transcript quantification and downstream differential expression analysis.

To further assess transcript structure representation, mapped reads were categorized as splice or non-splice reads. Splice reads comprised 36.76–44.06% of the aligned reads, while 50.29–58.02% were classified as non-splice reads, indicating the presence of well-represented mature transcripts in the sequencing libraries.

Exonic regions accounted for 95.69–96.85% of the uniquely mapped reads across all samples. Only minor proportions of reads were aligned to intronic (2.17–3.21%) and intergenic regions (0.95–1.18%). The dominance of exonic reads and the consistent distribution pattern among all treatment groups indicate high RNA integrity, effective mRNA enrichment, and minimal background or genomic contamination.

### 3.3. Comparative Quality and Consistency Analyses of Gene Expression Across All RNA Samples

To assess the quality, consistency and global characteristics of the RNA-seq data, gene expression distributions, sample correlations, and principal component analysis (PCA) were performed using log_2_(FPKM+1)–normalized expression values for all samples. Figures for the data in this subsection are available as Supplementary Data. The overall distribution of gene expression levels across all samples was examined using boxplots, FPKM density distribution curves (kernel density estimation), and violin plots based on log_2_(FPKM+1) values. Boxplot analysis showed highly comparable median expression levels and interquartile ranges among biological replicates within each treatment group (Supplementary Data). The similarity in distribution patterns across samples indicates effective normalization and minimal technical variation. No extreme deviations or outlier samples were observed, suggesting consistent sequencing depth and data quality across all libraries. The FPKM density distribution curves revealed high data concordance among all samples (Figure S1). The substantial overlap of density curves among ABZ, IVM, PZQ and DMSO samples indicates comparable gene expression distributions. Violin plots further illustrated the distribution and variability of gene expression levels across samples (Figure S2). The shapes of the violins were largely consistent among biological replicates and treatment groups, with similar central tendencies and spread. These results confirm uniform expression distributions and low intra-group variability.

Pearson correlation analysis was performed to evaluate the similarity of gene expression profiles among all samples (Figure S3). High correlation coefficients were observed across all pairwise comparisons, with R^2^ values ranging from 0.921 to 0.986. Biological replicates within the same treatment group exhibited high correlations with R^2^ values ≥ 0.94, indicating reproducibility and minimal experimental variation. Comparisons between treatment groups also showed high correlation values, reflecting overall transcriptomic similarity. Moreover, ivermectin-treated samples showed slightly lower correlation values when compared with the DMSO control (R^2^ values ≥ 0.921), suggesting greater transcriptomic difference relative to other treatments. In contrast, praziquantel- and albendazole-treated samples showed more similar correlation patterns to the control group with R^2^ values ≥ 0.943 and 0.949, respectively, indicating comparatively milder gene expression changes.

Principal component analysis (PCA) was conducted to visualize global transcriptional variation and sample clustering patterns (Figure S4). Biological replicates from the same treatment group clustered closely together, indicating high intra-group consistency. Ivermectin-treated samples exhibited the most distinct separation from DMSO, indicating a high global transcriptional response. Albendazole-treated samples formed a separate but closer cluster, suggesting a moderate effect. In contrast, praziquantel-treated samples showed partial overlap with controls, indicating its relatively limited impact on gene expression.

Taken together, the gene expression distribution analyses, correlation assessment, and PCA consistently demonstrate that the RNA-seq data are of high quality, well normalized, and reproducible across biological replicates. The observed clustering and separation patterns indicate distinct transcriptional responses to anthelmintic drug treatments, particularly ivermectin, providing a strong foundation for downstream differential gene expression and functional enrichment analyses.

### 3.4. Differential Gene Expression Analysis of Anthelminthic Drug-Treated FHs 74 Int Cells

**Figure 2:**
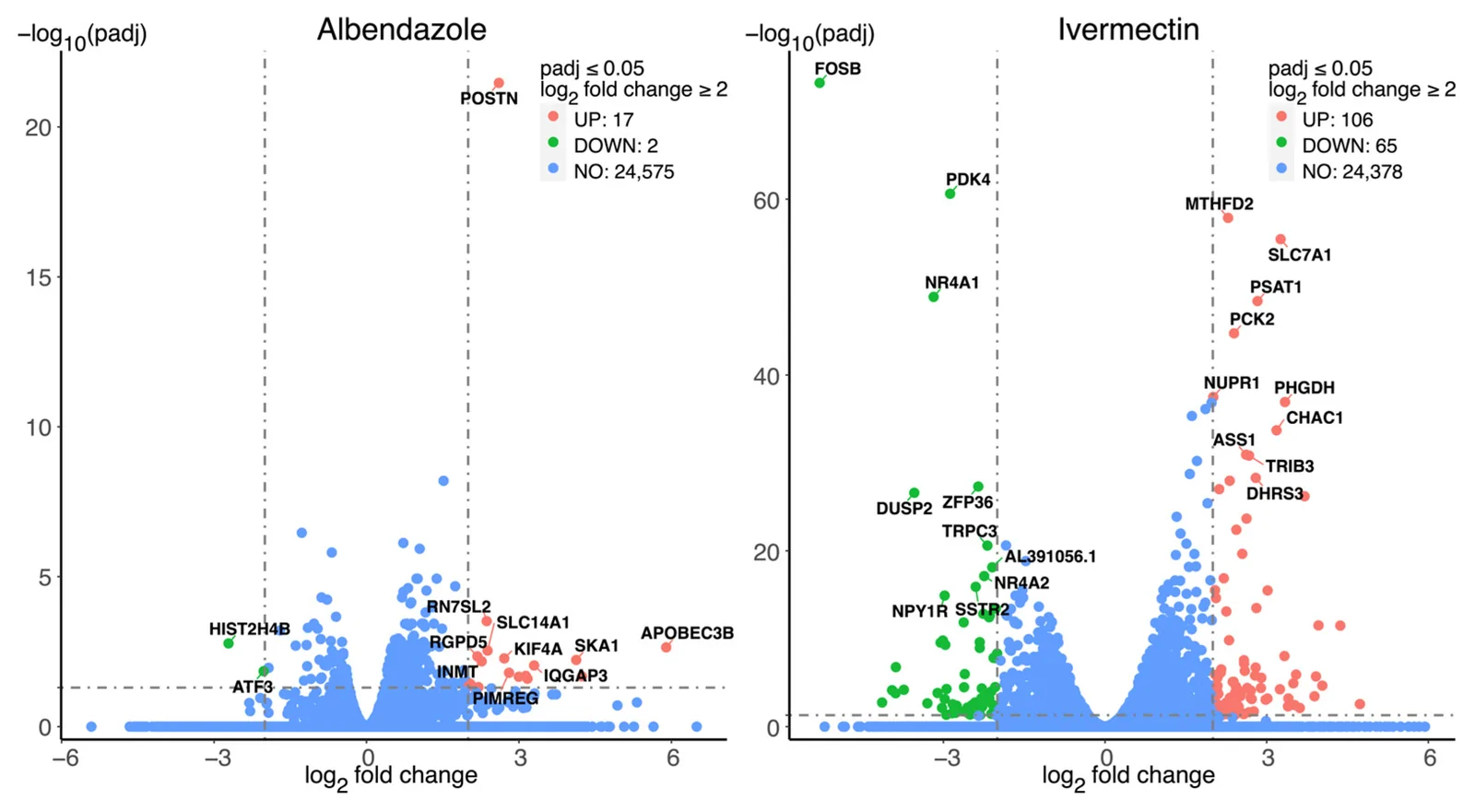
Volcano plots illustrating differential gene expression in FHs 74 Int cells treated with 1 µM albendazole and 15 µM ivermectin for 24 h compared to the DMSO control. Significant DEGs were identified using DESeq2 with a cutoff of padj ≤ 0.05 and log_2_ fold change ≥ 2. The top (padj) up/down DEGs are indicated by name. Dashed lines indicate the cutoff values.

Clean reads mapped to the human GRCh38 reference genome were quantified at the gene level and differential expression analysis was conducted using the DESeq2 statistical framework (Tables S1–S3). Genes with a padj ≤ 0.05 and an absolute log_2_ fold change ≥ 2 were considered differentially expressed. Treatment of FHs 74 Int cells with 1 µM albendazole for 24 h resulted in moderate transcriptional changes compared to the untreated control. Using the mentioned cutoff, 19 DEGs were identified in a total of 24,575 transcripts, including 17 up-regulated and 2 down-regulated genes (Table S4). Volcano plot visualization demonstrated a limited number of genes with large expression changes, suggesting a mild effect of albendazole on the gene expression profile of FHs 74 Int cells under these experimental conditions (Figure 2, Table 3).

Treatment with 15 µM ivermectin for 24 h induced larger transcriptomic changes. In a total of 24,378 transcripts, 171 DEGs were identified using the mentioned cutoff with 106 up-regulated and 65 down-regulated genes (Tables S5 and S6). Volcano plot analysis revealed a wide distribution of genes with low padj values, indicating a strong transcriptional response to ivermectin treatment (Figure 2, Table 3). The large number of DEGs suggested that ivermectin had a significant impact on gene regulatory processes in FHs 74 Int cells.

Treatment with 1000 µM praziquantel for 24 h had the least effect. Using the same cutoff criteria, 0 DEGs were identified in a total of 24,371 transcripts (Table 3). Even at log_2_ fold change ≥ 0 no DEGs were detected at padj ≤ 0.05. The low magnitude of expression changes suggested that praziquantel had limited effects on host cell transcription (Figure 2, Table 3). These findings were consistent with the absence of cytotoxic effects observed in the cell viability assays.

### 3.5. Gene Ontology Enrichment Analysis of Anthelminthic Drug-Treated FHs 74 Int Cells

Gene Ontology (GO) enrichment analysis was performed at https://functionome.geneontology.org/ (accessed on 28 July 2026) to explore the biological significance of DEGs identified at a cutoff padj ≤ 0.05 and log_2_ fold change ≥ 2 (Tables S4–S6). Enriched GO terms were classified into the three main categories: Biological Process (BP), Cellular Component (CC), and Molecular Function (MF).

In cells treated with 1 µM albendazole for 24 h, GO enrichment analysis of upregulated genes showed strong enrichment in BP related to mitotic cell cycle and chromosome segregation (Table 4). CC analysis revealed significant enrichment in structures required for mitosis, including the centromeric region, kinetochore, mitotic spindle, and condensed chromosome (Table 4). Molecular function analysis did not yield statistically significant results. Due to the small number, no enrichment analysis was possible for the two downregulated DEGs, HIST2H4B (histone cluster 2 H4 family member b) and ATF3 (activating transcription factor 3).

In cells treated with ivermectin, upregulated genes were strongly associated with BP related to biosynthesis of amino acids while no statistically significant results were found for CC and MF (Table 5).

In contrast, downregulated genes were primarily associated with BP related to cellular responses and metabolic processes related to hormones and drugs indicating cellular stress and changed metabolism (Table 6). No statistically significant results were found for CC.

GO enrichment analysis could not be performed for DEGs of cells treated with 1 mM praziquantel due to the fact that none had passed the padj ≤ 0.05 cutoff (Table 3).

### 3.6. Detection of Gene Interactions of Anthelminthic Drug-Treated FHs 74 Int Cells by STRING Analysis

**Figure 3:**
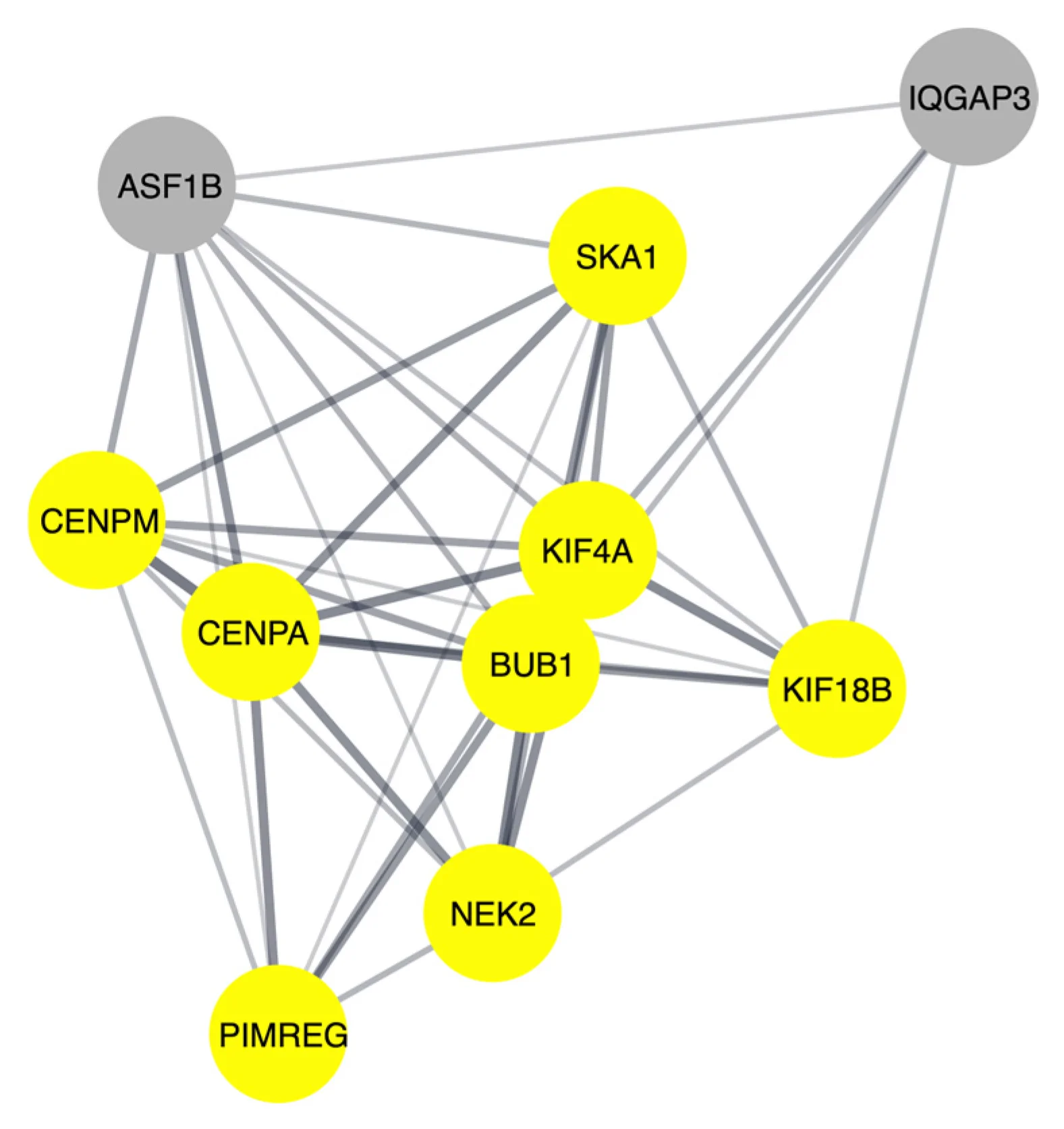
Largest DEG subnetwork at cutoff: log_2_ fold change ≥ 2, padj ≤ 0.05 of albendazole treated cells obtained in Cytoscape with stringApp. The eight upregulated DEGs contained in the most significant STRING cluster “Mixed, incl. Mitotic Spindle Checkpoint, and Mitotic sister chromatid segregation” (CL:6596) are shown in yellow. The remaining two DEGs of the subnetwork are shown in the default gray color. Stronger interactions of proteins are represented by thicker lines.

The STRING database was used through the Cytoscape application to investigate possible interactions and functions of the differentially regulated genes. The 17 up-regulated DEGs (Table S4) passing the cutoff criteria log_2_ fold change ≥ 2, padj ≤ 0.05 were used for analysis. The largest subnetwork contained 10 DEGs (Figure 3) and the most significant STRING cluster of those, CL:6596, was termed “Mixed, incl. Mitotic Spindle Checkpoint, and Mitotic sister chromatid segregation” with an FDR value of 3.05 × 10^−12^ (Table S7). It contained the eight upregulated DEGs shown in Table 7. Using a less stringent log_2_ fold change ≥ 1, *p*-value ≤ 0.05 STRING cluster CL:6596 contained 46 DEGs, FDR 5.82 × 10^−56^.

**Figure 4:**
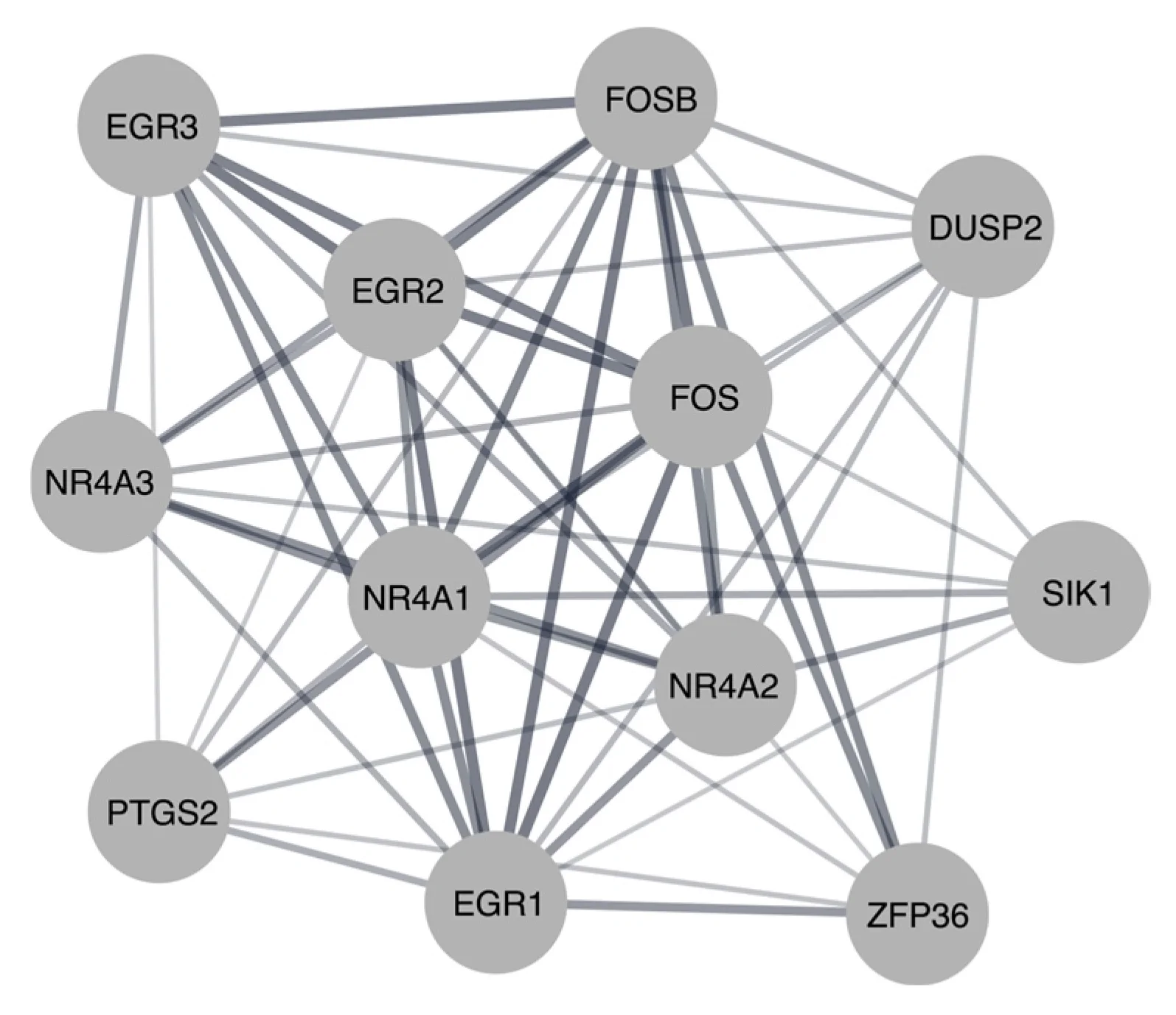
MCODE cluster 1 comprising the 12 most densely connected downregulated proteins of the largest subnetwork of DEGs (cutoff: log_2_ fold change ≥ 2, padj ≤ 0.05) of ivermectin treated cells obtained in Cytoscape with stringApp and MCODE (complete enrichment data available in Table S8). Stronger interactions of proteins are represented by thicker lines.

In the case of ivermectin, at cutoff criteria of log_2_ fold change ≥ 2 and padj ≤ 0.05, 106 upregulated and 65 downregulated DEGs were left (Tables S5 and S6), and 135 of them were found in STRING database with 232 edges between them. The largest subnetwork contained 86 DEGs with 229 edges and the largest MCODE cluster of it contained 12 downregulated DEGs with 55 edges (Figure 4). All proteins were found in the nucleus, with 11 involved in the regulation of gene expression in response to cellular stimulus and FOS as the root element of the cluster (Table 8, Table S8). Eight of the DEGs are members of the three protein families FOS, NR4A, and ERG whose downregulation has been associated with reduced cell proliferation. The five DEGs FOSB, NR4A1, ZFP36, DUSP2, NR4A2 are the top 1, 3, 4, 5, and 8 downregulated DEGs (Table S6).

**Figure 5:**
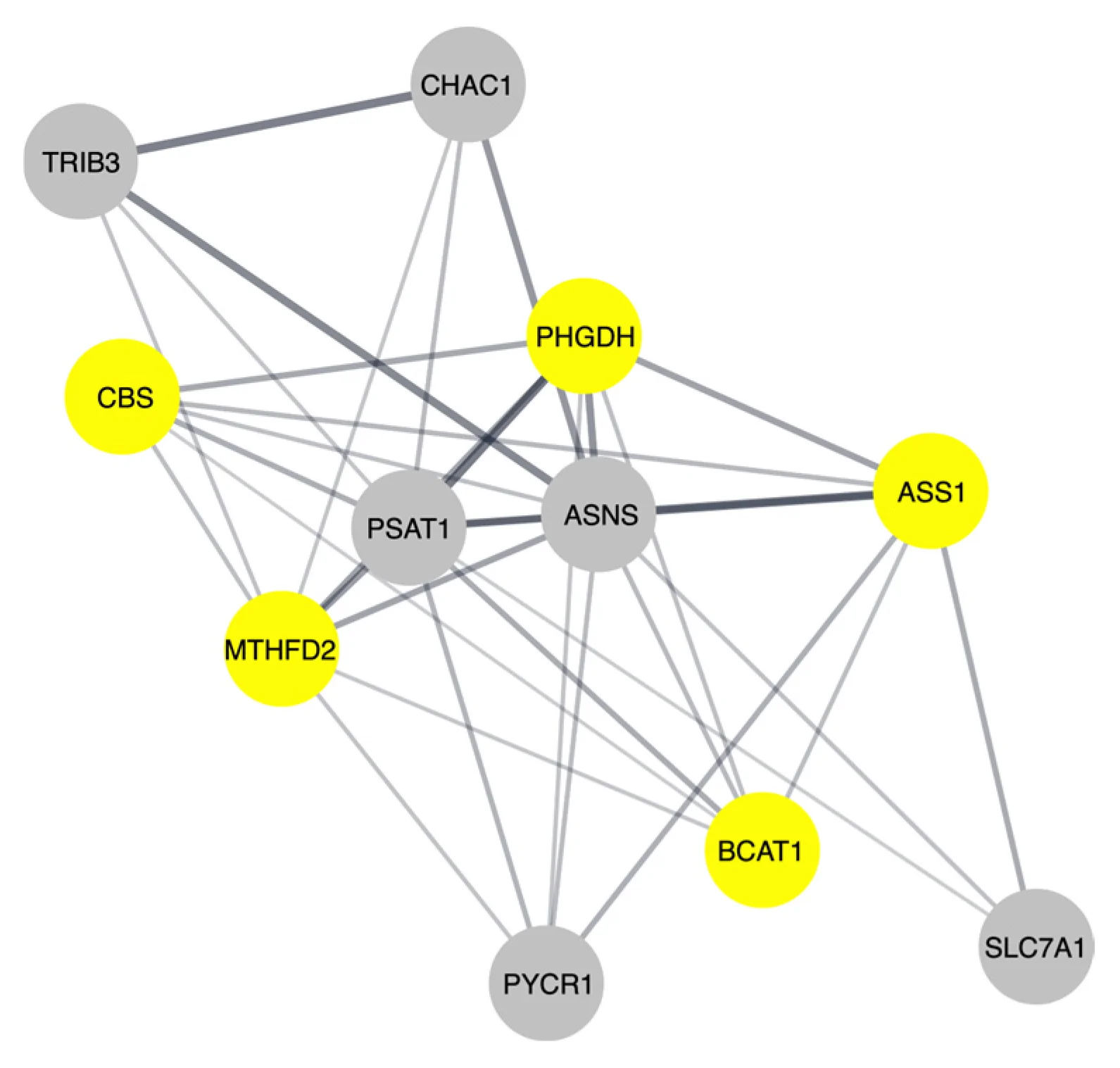
Combined MCODE cluster 2 (yellow circles) and 3 (gray circles) of the largest subnetwork of DEGs (cutoff: log_2_ fold change ≥ 2, padj ≤ 0.05) of ivermectin treated cells obtained in Cytoscape with stringApp and MCODE (complete enrichment data is available in Table S9). Stronger interactions of proteins are represented by thicker lines.

The next two MCODE clusters were actually intertwined and contained only upregulated DEGS, including 7 of the top 10 upregulated DEGs (rank 1, 2, 3, 6, 7, 8, 9, Table S5). They are mostly metabolic enzymes with MTHFD2, PHGDH, ASS1, BCAT1, CBS (rank 1, 6, 8, 15, 83) in cluster 2 and SLC7A1, PSAT1, CHAC1, TRIB3, PYCR1, ASNS (rank 2, 3, 7, 9, 16, 36) in cluster 3 (Figure 5). Their functional focus is on the biosynthesis of amino acids (Table 9, Table S9). The remaining two proteins in the top 10 upregulated DEGs are PCK2 (phosphoenolpyruvate carboxykinase 2, log_2_ fold change 8.221 × 10^−49^, padj 1.821 × 10^−45^) and NUPR1 (nuclear protein 1, log_2_ fold change 1.632 × 10^−41^, padj 3.163 × 10^−38^).

For praziquantel it was not possible to find meaningful networks or functions as the cells obviously did not react to this drug and even at log_2_ fold change ≥ 0 not a single DEG passed padj ≤ 0.05.

## 4. Discussion

In this study, FHs 74 Int, a primary human cell line of the small intestine, was selected for analysis of the effects of three common anthelminthic drugs, albendazole, ivermectin and praziquantel. The effects on the cells were observed through detection of differentially expressed genes by transcriptome analysis following 24 h of exposure of the cells to the drugs. It is assumed that FHs 74 Int cells represent normal diploid cells with a standard genome. The cell line is of fetal origin and the cells expressed a rich transcriptome with more than 24,000 different transcripts in each of the drug-treated and control samples. The drugs are not or minimally soluble in water and had to be solubilized in DMSO. Therefore, the incubation medium contained 0.5% DMSO including for the control group. It cannot be excluded that the presence of DMSO affected transcriptional changes caused by the drugs. The selected drug concentrations of 1 µM albendazole, 15 µM ivermectin and 1 mM praziquantel caused marginal cytotoxicity after a 24-h incubation (Figure 1). Compared to reported plasma concentrations in drug-treated patients, these concentrations are 2–10× higher for albendazole, 200–400× higher for ivermectin, and 500–3000× higher for praziquantel [27]. However, similar and higher concentrations have been used for functional analysis in in vitro assays of mammalian cells. Albendazole at 10–500 µM concentration was used to test its uptake by rat enterocytes [28]. Ivermectin, effective at nanomolar concentrations against invertebrate glutamate-gated Cl-channels, required micromolar concentrations to be effective against mammalian targets like the P2X receptor channels [29,30]. Praziquantel was used at concentrations between 2.5 and 160 µM to study cytotoxicity in several human cell lines [31].

Albendazole sulfoxide is the active metabolite of albendazole and is mostly generated in the liver. However, intestinal uptake and conversion of albendazole into albendazole sulfoxide have been demonstrated [32,33]. The objective of this study was to detect and classify transcriptional changes that are triggered by the three drugs in whatever form they are present in the medium or taken up by the cells. This should allow for the evaluation of their potential negative effects on human cells or their application for treatment of other diseases by knowing which regulatory pathways they interfere with. The three drugs had very different effects on the cells. First of all, praziquantel had no negative effect on FHs 74 Int cells. Even at the highest concentration of 1 mM that could be achieved at 0.5% DMSO, the cells survived for 72 h and the transcriptome changes after 24-h exposure were minimal and not meaningful. The result is consistent with previous studies showing that praziquantel has low toxicity in mammalian cells and mainly targets parasites through calcium-related mechanisms which may explain the weak transcriptional response observed in FHs 74 Int cells [31,34,35].

In contrast, albendazole and ivermectin not only caused cytotoxicity at micromolar concentrations (Figure 1) but also transcriptional changes that were highly different for the two drugs (Figure 2, Table 3). It should be noted that it was not possible to extract RNA at sufficient quality from cells treated with 10 µM albendazole, though how the drug interfered with the extraction process remains unknown. However, even at 1 µM albendazole a substantial number of differentially expressed genes were observed (Table 3). Analysis of the functions of these genes and clustering of them into potential networks found that a substantial number of them were involved in biological processes that connect to the major described action of albendazole as an inhibitor of microtubule formation (Table 4 and Table 7). The eight upregulated DEGs remaining at log_2_ fold change ≥ 2/padj ≤ 0.05 (Figure 3, Table 7) suggested that the cells tried to resolve processes concerning mitotic spindle, chromosome segregation at the centromere/kinetochore during meta/anaphase of mitosis. This might be in consequence of the disruption of microtubule formation by the drug and suggests that it does not only affect helminth microtubules but also affects microtubule formation in human cells. The results are consistent with previous experimental studies which demonstrated that albendazole disrupts microtubule formation and arrests mammalian cells in the G2/M phase of the cell cycle [36,37,38]. Finally, when observing the padj values it should be noted that the transcriptional response was moderate for albendazole when compared to ivermectin.

The third drug, ivermectin, caused a stronger transcriptional response. At cutoff padj ≤ 0.05, log_2_ fold change ≥ 2 a substantially larger number of genes was differentially regulated (Table 3). It seemed that ivermectin treatment had broader consequences on gene expression because the up/downregulated DEGs classified in GO biological processes affected various kinds of cell responses, cell regulation and cell signaling (Table 5 and Table 6). These findings were consistent with previous studies in mammalian cells demonstrating that ivermectin induced several pathways such as cell cycle arrest, apoptosis, and oxidative stress, and modulates key signaling pathways including Akt/mTOR and MAPK, leading to disruption of cellular homeostasis [16,17,39,40,41]. However, a clear pattern emerged after focusing on the most significantly up/downregulated DEGs. Many of them were found in the three top MCODE clusters which comprised downregulated, nuclear proteins in cluster 1 and upregulated metabolic enzymes in cluster 2 and 3 (Table 8 and Table 9). In respect to their function, the downregulated nuclear proteins suggested inhibition of cell proliferation while the upregulated metabolic enzymes suggested support of cell survival and growth. Generally speaking, ivermectin treatment caused cellular stress that triggered a metabolic reprogramming aiming at cell survival and growth and not at cell proliferation. In recent studies, these clustered proteins have been implicated in cancer and as master switches in disease including the remaining two proteins in the top 10 upregulated DEGs, PCK2 (phosphoenolpyruvate carboxykinase 2) and NUPR1 (nuclear protein 1) [42,43,44,45,46]. However, there was no apparent direct connection between ivermectin and any of these proteins.

Regarding limitations, it should be noted that these results, while consistent with previous research, would still need to be further investigated and confirmed. What happens at longer exposure times? Does albendazole block the cell cycle, or will the cells continue to proliferate? Can the genes that were found differentially regulated after ivermectin treatment be confirmed by qPCR, and by which direct action(s) does the drug stimulate these changes? Furthermore, FHs 74 Int is a fetal cell line; the results might be different in adult stage enterocytes.

In conclusion, this research found networks of differentially regulated genes in FHs 74 Int enterocytes upon exposure to high concentrations of albendazole and ivermectin. The data should help to explain the cytotoxicity of these drugs at concentrations that exeed standard prescribed dosages. Remarkably, praziquantel at 1 mM concentration affected neither cell viability nor gene expression of the tested cells. As discussed above, this supports previous studies that found no effect of praziquantel on cell viability in a small number of other human cell types. Furthermore, praziquantel was found to be highly tolerated in animals; for example rats treated with a daily dose of 1 g/kg for four weeks showed no damage and the oral LD50 dose was 2.84 g/kg [47].

## Figures and Tables

**Table 1:** Results of the quality control of the used RNA samples.

Sample	Concentration (ng/µL)	Total Amount (µg)	RIN ^1^
1 µM albendazole treated FHs 74 int cells (ABZ)			
ABZ1	14.00	0.504	8.80
ABZ2	14.00	0.504	9.90
ABZ3	15.00	0.420	9.20
15 µM ivermectin treated FHs 74 int cells (IVM)			
IVM1	13.00	0.455	6.90
IVM2	16.00	0.576	8.70
IVM3	12.00	0.432	8.40
1000 µM praziquantel treated FHs 74 int cells (PZQ)			
PZQ1	12.00	0.408	7.70
PZQ2	18.00	0.648	8.10
PZQ3	11.00	0.407	8.20
0.5% DMSO treated FHs 74 int cells (DMSO)			
DMSO1	20.00	0.720	8.20
DMSO2	17.00	0.612	9.00
DMSO3	18.00	0.648	7.50

^1^ RIN = RNA Integrity Number.

**Table 2:** Summary of read mapping against human reference genome GRCh38.

Sample	Total Clean Reads	Total Mapped Reads (%)	Uniquely Mapped Reads (%)	Multiple Mapped Reads (%)	Splice Reads (%)	Non-Splice Reads (%)
ABZ1	5.69$\;\times \;10^{7}$	96.33	93.96	2.37	40.53	53.43
ABZ2	6.58$\;\times \;10^{7}$	95.93	93.52	2.41	39.19	54.33
ABZ3	4.04$\;\times \;10^{7}$	96.15	93.74	2.41	40.83	52.92
IVM1	4.80$\;\times \;10^{7}$	95.81	93.41	2.40	40.29	53.12
IVM2	4.53$\;\times \;10^{7}$	96.63	94.35	2.27	44.06	50.29
IVM3	4.43$\;\times \;10^{7}$	96.10	93.72	2.39	39.67	54.04
PZQ1	4.38$\;\times \;10^{7}$	96.15	93.83	2.32	40.09	53.74
PZQ2	4.20$\;\times \;10^{7}$	96.83	94.77	2.06	36.76	58.02
PZQ3	4.34$\;\times \;10^{7}$	96.73	94.61	2.12	41.63	52.99
DMSO1	5.69$\;\times \;10^{7}$	96.84	94.66	2.19	40.38	54.28
DMSO2	4.49$\;\times \;10^{7}$	97.11	95.04	2.07	38.79	56.25
DMSO3	5.48$\;\times \;10^{7}$	96.58	94.28	2.30	38.74	55.54

**Table 3:** Number of differentially expressed genes at different cutoff values in FHs 74 Int cells treated with 1 µM albendazole, 15 µM ivermectin, or 1 mM praziquantel for 24 h compared to the DMSO control.

Cutoff	Albendazole		Ivermectin		Praziquantel	
	Up	Down	Up	Down	Up	Down
*p*-value ≤ 0.05, log_2_ fold change ≥ 0	1359	877	2422	2653	188	139
padj ≤ 0.05, log_2_ fold change ≥ 0	271	84	1484	1585	0	0
*p*-value ≤ 0.05, log_2_ fold change ≥ 1	284	95	731	376	69	48
padj ≤ 0.05, log_2_ fold change ≥ 1	63	12	483	263	0	0
*p*-value ≤ 0.05, log_2_ fold change ≥ 2	102	39	228	114	42	20
padj ≤ 0.05, log_2_ fold change ≥ 2	17	2	106	65	0	0

**Table 4:** Results of PAN-GO version 2.0 overrepresentation test for upregulated DEGs at log_2_ fold change ≥ 2, padj ≤ 0.05 of albendazole treated cells (cutoff: FDR 0.05).

**Biological Process**	**GO-ID**	**Fold Enrichment**	**FDR**
chromosome segregation	GO:0007059	45.94	7.68 × 10^−5^
mitotic cell cycle process	GO:1903047	38.21	2.11 × 10^−5^
mitotic cell cycle	GO:0000278	36.16	2.50 × 10^−6^
cell cycle process	GO:0022402	23.69	1.58 × 10^−5^
cell cycle	GO:0007049	21.29	2.48 × 10^−5^
**Cellular Component**	**GO-ID**	**Fold Enrichment**	**FDR**
spindle microtubule	GO:0005876	>100	1.93 × 10^−2^
mitotic spindle	GO:0072686	75.66	4.19 × 10^−2^
kinetochore	GO:0000776	65.40	1.63 × 10^−2^
condensed chromosome, centromeric region	GO:0000779	61.25	9.96 × 10^−3^
chromosome, centromeric region	GO:0000775	51.45	1.12 × 10^−2^
chromosomal region	GO:0098687	43.36	1.41 × 10^−2^
condensed chromosome	GO:0000793	36.40	1.35 × 10^−2^
supramolecular complex	GO:0099080	10.54	1.25 × 10^−2^
intracellular membraneless organelle	GO:0043232	5.04	1.53 × 10^−2^
membraneless organelle	GO:0043228	5.04	1.28 × 10^−2^

**Table 5:** Results of PAN-GO version 2.0 overrepresentation test for upregulated DEGs at log_2_ fold change ≥ 2, padj ≤ 0.05 of ivermectin treated cells (cutoff FDR 0.05).

Biological Process	GO_ID	Fold Enrichment	FDR
serine family amino acid biosynthetic process	GO:0009070	60.71	2.28 × 10^−2^
alpha-amino acid biosynthetic process	GO:1901607	28.76	3.95 × 10^−4^
proteinogenic amino acid biosynthetic process	GO:0170038	27.59	2.20 × 10^−3^
amino acid biosynthetic process	GO:0008652	26.02	3.68 × 10^−4^
L-amino acid biosynthetic process	GO:0170034	24.28	2.43 × 10^−2^
proteinogenic amino acid metabolic process	GO:0170039	11.50	1.93 × 10^−2^
alpha-amino acid metabolic process	GO:1901605	10.31	2.57 × 10^−2^
carboxylic acid biosynthetic process	GO:0046394	9.42	3.75 × 10^−2^
organic acid biosynthetic process	GO:0016053	9.11	4.03 × 10^−2^

**Table 6:** Results of PAN-GO Version 2.0 overrepresentation test for downregulated DEGs at log_2_ fold change ≥ 2, padj ≤ 0.05 of ivermectin treated cells (cutoff FDR 0.05).

**Biological Process**	**GO-ID**	**Fold Enrichment**	**FDR**
cellular response to corticotropin-releasing hormone stimulus	GO:0071376	>100	2.48 × 10^−4^
response to corticotropin-releasing hormone	GO:0043435	>100	1.24 × 10^−4^
polyketide metabolic process	GO:0030638	>100	4.56 × 10^−2^
doxorubicin metabolic process	GO:0044598	>100	4.14 × 10^−2^
daunorubicin metabolic process	GO:0044597	>100	3.80 × 10^−2^
progesterone metabolic process	GO:0042448	>100	4.21 × 10^−2^
prostaglandin metabolic process	GO:0006693	63.91	2.93 × 10^−2^
prostanoid metabolic process	GO:0006692	63.91	2.20 × 10^−2^
cellular response to peptide hormone stimulus	GO:0071375	17.81	3.72 × 10^−2^
response to peptide hormone	GO:0043434	15.30	4.71 × 10^−2^
cellular response to nitrogen compound	GO:1901699	11.56	3.86 × 10^−2^
cellular response to hormone stimulus	GO:0032870	10.21	4.74 × 10^−2^
cellular response to oxygen-containing compound	GO:1901701	9.22	4.68 × 10^−2^
cellular response to endogenous stimulus	GO:0071495	8.19	3.19 × 10^−2^
response to oxygen-containing compound	GO:1901700	7.95	4.37 × 10^−2^
response to endogenous stimulus	GO:0009719	7.40	5.05 × 10^−2^
multicellular organism development	GO:0007275	3.61	4.27 × 10^−2^
system development	GO:0048731	3.59	4.57 × 10^−2^
cellular developmental process	GO:0048869	3.47	4.81 × 10^−2^
cell differentiation	GO:0030154	3.47	4.60 × 10^−2^
anatomical structure development	GO:0048856	3.01	4.40 × 10^−2^
multicellular organismal process	GO:0032501	2.86	4.33 × 10^−2^
**Molecular Function**	**GO-ID**	**Fold Enrichment**	**FDR**
nuclear glucocorticoid receptor binding	GO:0035259	>100	4.73 × 10^−4^

**Table 7:** The top eight upregulated DEGs of albendazole treated cells contained in STRING cluster “Mixed, incl. Mitotic Spindle Checkpoint, and Mitotic sister chromatid segregation” (CL:6596) as obtained in Cytoscape with stringApp.

log_2_FC	padj	ID	Description (Source: StringDB)
2.711	0.005	KIF4A	Chromosome-associated kinesin KIF4A; Motor protein that translocates PRC1 to the plus ends of interdigitating spindle microtubules during the metaphase to anaphase transition.
4.126	0.006	SKA1	Spindle and kinetochore-associated protein 1; Component of the SKA1 complex, a microtubule-binding subcomplex of the outer kinetochore that is essential for proper chromosome segregation.
2.805	0.016	PIMREG	Protein PIMREG; During mitosis, may play a role in the control of metaphase- to-anaphase transition.
3.134	0.020	KIF18B	Kinesin-like protein KIF18B; In complex with KIF2C, constitutes the major microtubule plus-end depolymerizing activity in mitotic cells.
4.240	0.021	CENPM	Centromere protein M; Component of the CENPA-NAC (nucleosome-associated) complex, a complex that plays a central role in assembly of kinetochore proteins, mitotic progression and chromosome segregation.
2.997	0.022	CENPA	Histone H3-like centromeric protein A; Histone H3-like nucleosomal protein that is specifically found in centromeric nucleosomes. Replaces conventional H3 in the nucleosome core of centromeric chromatin at the inner plate of the kinetochore.
3.168	0.025	NEK2	Serine/threonine-protein kinase Nek2; Protein kinase which is involved in the control of centrosome separation and bipolar spindle formation in mitotic cells and chromatin condensation in meiotic cells.
2.202	0.048	BUB1	Mitotic checkpoint serine/threonine-protein kinase BUB1; Serine/threonine-protein kinase that performs 2 crucial functions during mitosis: it is essential for spindle-assembly checkpoint signaling and for correct chromosome alignment.

**Table 8:** The twelve downregulated members of MCODE cluster 1 of ivermectin treated cells shown in Figure 4 (complete enrichment data is available in Table S8).

log_2_FC	padj	ID	Description (Source: StringDB)
−5.296	5.953 × 10^−74^	FOSB	Protein fosB; FosB interacts with Jun proteins enhancing their DNA binding activity.
−3.181	1.314 × 10^−49^	NR4A1	Nuclear receptor subfamily 4 group A member 1; Orphan nuclear receptor.
−2.353	4.892 × 10^−28^	ZFP36	mRNA decay activator protein ZFP36; Zinc-finger RNA-binding protein that destabilizes several cytoplasmic AU-rich element (ARE)-containing mRNA transcripts by promoting their poly(A) tail removal or deadenylation, and hence provide a mechanism for attenuating protein synthesis.
−3.540	2.508 × 10^−27^	DUSP2	Dual specificity protein phosphatase 2; Regulates mitogenic signal transduction by dephosphorylating both Thr and Tyr residues on MAP kinases ERK1 and ERK2.
−2.242	7.478 × 10^−18^	NR4A2	Nuclear receptor subfamily 4 group A member 2; Transcriptional regulator which is important for the differentiation and maintenance of meso-diencephalic dopaminergic (mdDA) neurons during development.
−3.882	1.698 × 10^−7^	FOS	Proto-oncogene c-Fos; Nuclear phosphoprotein which forms a tight but non-covalently linked complex with the JUN/AP-1 transcription factor.
−2.113	9.997 × 10^−5^	EGR1	Early growth response protein 1; Transcriptional regulator.
−3.108	1.481 × 10^−4^	EGR2	E3 SUMO-protein ligase EGR2; Sequence-specific DNA-binding transcription factor.
−2.146	6.272 × 10^−4^	EGR3	Early growth response protein 3; Probable transcription factor involved in muscle spindle development; Belongs to the EGR C2H2-type zinc-finger protein family.
−3.297	2.212 × 10^−3^	SIK1	Serine/threonine-protein kinase SIK1; Serine/threonine-protein kinase involved in various processes such as cell cycle regulation, gluconeogenesis and lipogenesis regulation, muscle growth and differentiation and tumor suppression.
−2.318	2.408 × 10^−3^	NR4A3	Nuclear receptor subfamily 4 group A member 3; Transcriptional activator that binds to regulatory elements in promoter regions in a cell- and response element (target)-specific manner.
−2.258	2.673 × 10^−3^	PTGS2	Prostaglandin G/H synthase 2; Converts arachidonate to prostaglandin H2 (PGH2), a committed step in prostanoid synthesis.

**Table 9:** The eleven members of MCODE cluster 2 and 3 of ivermectin treated cells shown in Figure 5 (complete enrichment data available in Table S9).

log_2_FC	padj	ID	Description (Source: StringDB)
2.284	1.363 × 10^−58^	MTHFD2	Bifunctional methylenetetrahydrofolate dehydrogenase/cyclohydrolase, mitochondrial.
3.259	3.511 × 10^−56^	SLC7A1	High affinity cationic amino acid transporter 1; High-affinity, low capacity permease involved in the transport of the cationic amino acids (arginine, lysine and ornithine) in non-hepatic tissues.
2.829	3.959 × 10^−49^	PSAT1	Phosphoserine aminotransferase; Catalyzes the reversible conversion of 3- phosphohydroxypyruvate to phosphoserine and of 3-hydroxy-2-oxo-4- phosphonooxybutanoate to phosphohydroxythreonine.
3.340	1.193 × 10^−37^	PHGDH	D-3-phosphoglycerate dehydrogenase; Catalyzes the reversible oxidation of 3-phospho-D-glycerate to 3-phosphonooxypyruvate, the first step of the phosphorylated L- serine biosynthesis pathway.
3.182	1.961 × 10^−34^	CHAC1	Glutathione-specific gamma-glutamylcyclotransferase 1; Catalyzes the cleavage of glutathione into 5-oxo-L-proline and a Cys-Gly dipeptide. Glutathione depletion is an important factor for apoptosis initiation and execution.
2.618	1.156 × 10^−31^	ASS1	Argininosuccinate synthase; Catalyzes the formation of arginosuccinate from aspartate, citrulline and ATP and together with ASL it is responsible for the biosynthesis of arginine in most body tissues.
2.673	1.491 × 10^−31^	TRIB3	Tribbles homolog 3; May bind directly to and mask the ‘Thr- 308’ phosphorylation site in AKT1. Binds to ATF4 and inhibits its transcriptional activation activity. Interacts with the NF-kappa-B transactivator p65 RELA and inhibits its phosphorylation and thus its transcriptional activation activity. Interacts with MAPK kinases and regulates activation of MAP kinases.
2.437	4.021 × 10^−23^	BCAT1	Branched-chain-amino-acid aminotransferase, cytosolic; Catalyzes the first reaction in the catabolism of the essential branched chain amino acids leucine, isoleucine, and valine.
2.547	2.199 × 10^−20^	PYCR1	Pyrroline-5-carboxylate reductase 1, mitochondrial; Housekeeping enzyme that catalyzes the last step in proline biosynthesis.
3.915	1.946 × 10^−6^	ASNS	Asparagine synthetase.
2.144	1.078 × 10^−2^	CBS	Cystathionine beta-synthase-like protein; Hydro-lyase catalyzing the first step of the transsulfuration pathway, where the hydroxyl group of L-serine is displaced by L-homocysteine in a beta-replacement reaction to form L-cystathionine, the precursor of L-cysteine.

## Data Availability

The original data presented in the study are openly available in the Sequence Read Archive (SRA) under BioProject accession PRJNA1483491.

## Supplementary Materials

The following supporting information can be downloaded at: https://www.mdpi.com/article/10.3390/tropicalmed11090257/s1, Figure S1: The FPKM density distribution curves of gene expression levels in drug-treated FHs 74 Int cells; Figure S2: Violin plot of gene expression level distribution in drug-treated FHs 74 Int cells; Figure S3: Pearson correlation heatmap based on gene expression profiles in drug-treated FHs 74 Int cells; Figure S4: Principal component analysis (PCA) of gene expression profiles in drug-treated FHs 74 Int cells; Table S1: Differentially expressed genes of albendazole-treated FHs 74 Int (log_2_ fold change ≥ 0, *p*-value ≤ 0.05); Table S2: Differentially expressed genes of ivermectin-treated FHs 74 Int (log_2_ fold change ≥ 0, *p*-value ≤ 0.05); Table S3: Differentially expressed genes of praziquantel-treated FHs 74 Int (log_2_ fold change ≥ 0, *p*-value ≤ 0.05); Table S4: The up/down-regulated DEGs at log_2_ fold change ≥ 2, padj ≤ 0.05 following 1 µM albendazole treatment for 24 h; Table S5: The upregulated DEGs at log_2_ fold change ≥ 2, padj ≤ 0.05 following 15 µM ivermectin treatment for 24 h; Table S6: The downregulated DEGs at log_2_ fold change ≥ 2, padj ≤ 0.05 following 15 µM ivermectin treatment for 24 h; Table S7: Cytoscape largest subnetwork functional enrichment analysis of DEGs from ABZ treated cells; Table S8: Cytoscape largest subnetwork MCODE cluster 1 functional enrichment analysis of IVM treated cells; Table S9: Cytoscape largest subnetwork MCODE cluster 2 and 3 functional enrichment analysis of IVM treated cells.

## Abbreviations

ABZ	Albendazole
IVM	Ivermectin
PZQ	Praziquantel
padj	adjusted *p*-value
GO	Gene Ontology
BP	Biological Process
CC	Cellular Component
MF	Molecular Function
