# Supplementary material for: Effects of Three Anthelmintics on the Transcriptome of FHs 74 Int Enterocytes
Source: Trop Med Infect Dis. 2026 Sep 10;11(9):257. doi: 10.3390/tropicalmed11090257 (PMC13611716; doi:10.3390/tropicalmed11090257)
Supplement: Supplementary file 1 [file tropicalmed-11-00257-s001.zip › Figure S1.pdf]

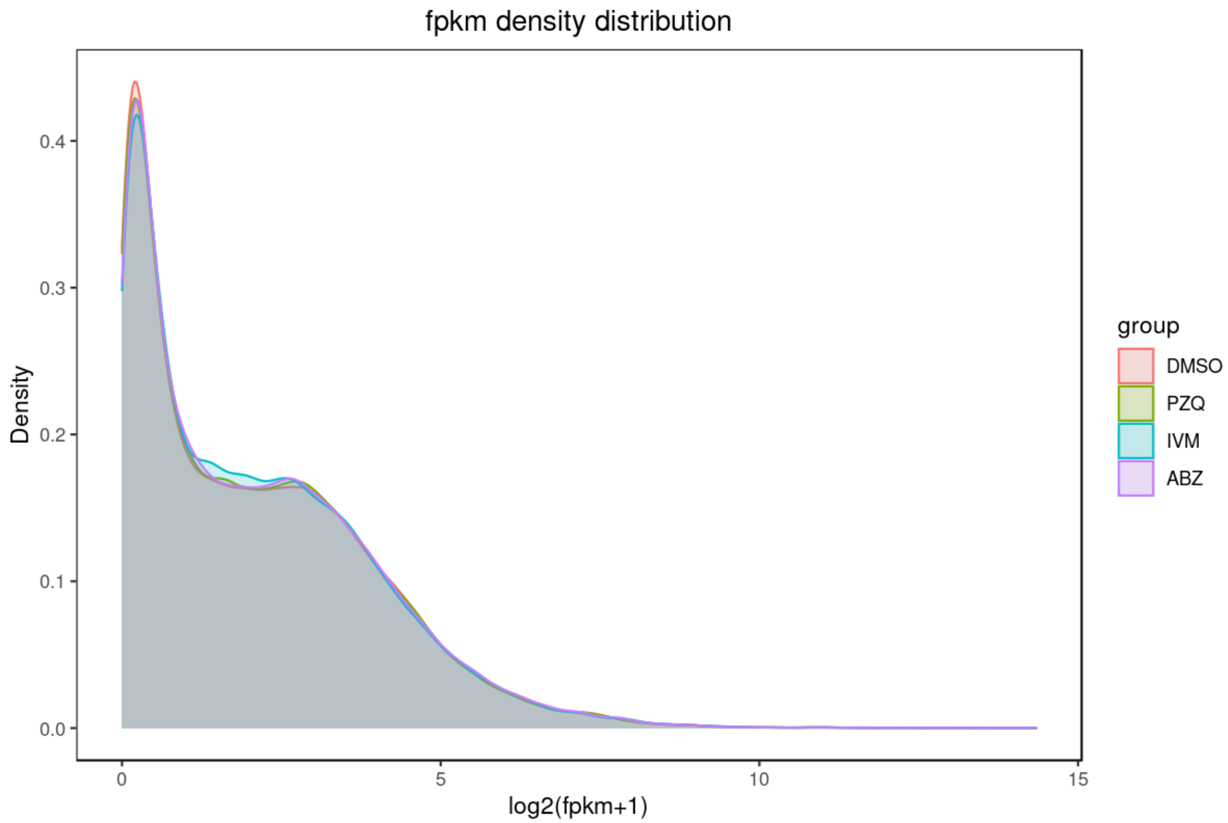

Figure S1. The FPKM density distribution curves of gene expression levels in drug-treated FHs 74 Int cells. FPKM density distribution curves illustrating the global distribution of gene expression levels across samples based on log-transformed FPKM values [$\log_2$(FPKM + 1)].
