# Supplementary material for: Effects of Three Anthelmintics on the Transcriptome of FHs 74 Int Enterocytes
Source: Trop Med Infect Dis. 2026 Sep 10;11(9):257. doi: 10.3390/tropicalmed11090257 (PMC13611716; doi:10.3390/tropicalmed11090257)
Supplement: Supplementary file 1 [file tropicalmed-11-00257-s001.zip › Figure S2.pdf]

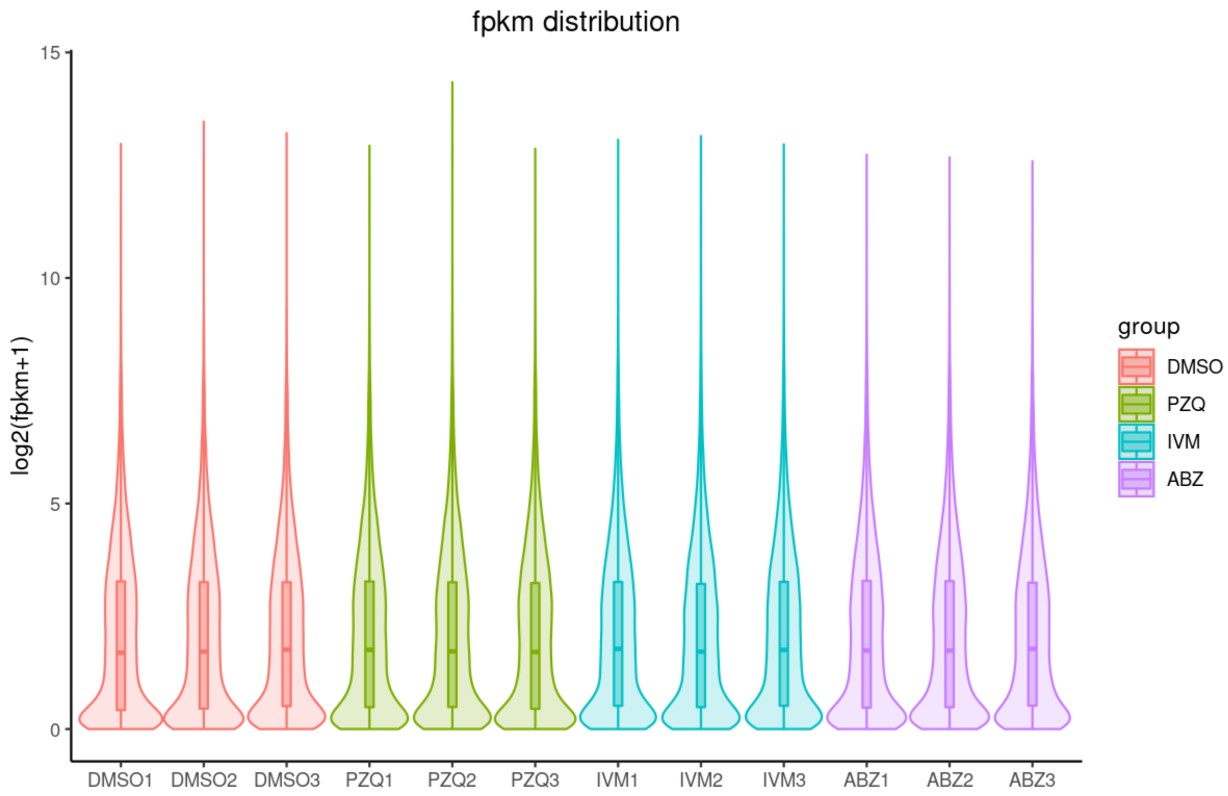

Figure S2. Violin plot of gene expression level distribution in drug-treated FHs 74 Int cells. Violin plots with embedded boxplots showing the distribution of gene expression levels across samples based on log-transformed FPKM values [$\log_2$(FPKM + 1)].
