# Supplementary material for: Effects of Three Anthelmintics on the Transcriptome of FHs 74 Int Enterocytes
Source: Trop Med Infect Dis. 2026 Sep 10;11(9):257. doi: 10.3390/tropicalmed11090257 (PMC13611716; doi:10.3390/tropicalmed11090257)
Supplement: Supplementary file 1 [file tropicalmed-11-00257-s001.zip › Figure S3.pdf]

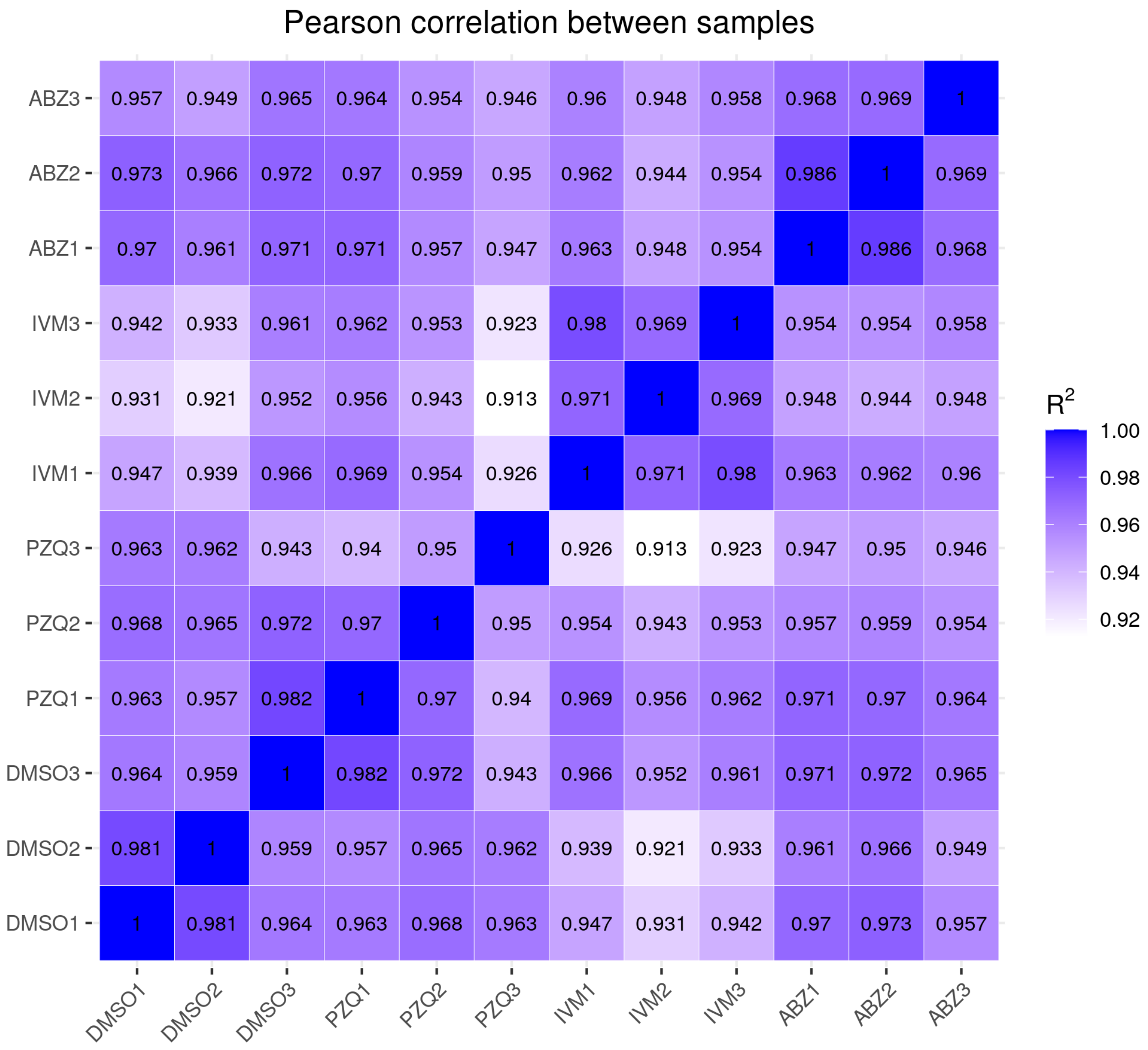

Figure S3. Pearson correlation heatmap based on gene expression profiles in drug-treated FHs 74 Int cells. Heatmap showing pairwise correlation coefficients between samples calculated from normalized gene expression values (FPKM). Higher correlation coefficients indicate greater similarity in gene expression profiles among samples, allowing assessment of reproducibility within biological replicates and differences between experimental groups.
