# Supplementary material for: Effects of Three Anthelmintics on the Transcriptome of FHs 74 Int Enterocytes
Source: Trop Med Infect Dis. 2026 Sep 10;11(9):257. doi: 10.3390/tropicalmed11090257 (PMC13611716; doi:10.3390/tropicalmed11090257)
Supplement: Supplementary file 1 [file tropicalmed-11-00257-s001.zip › Figure S4.pdf]

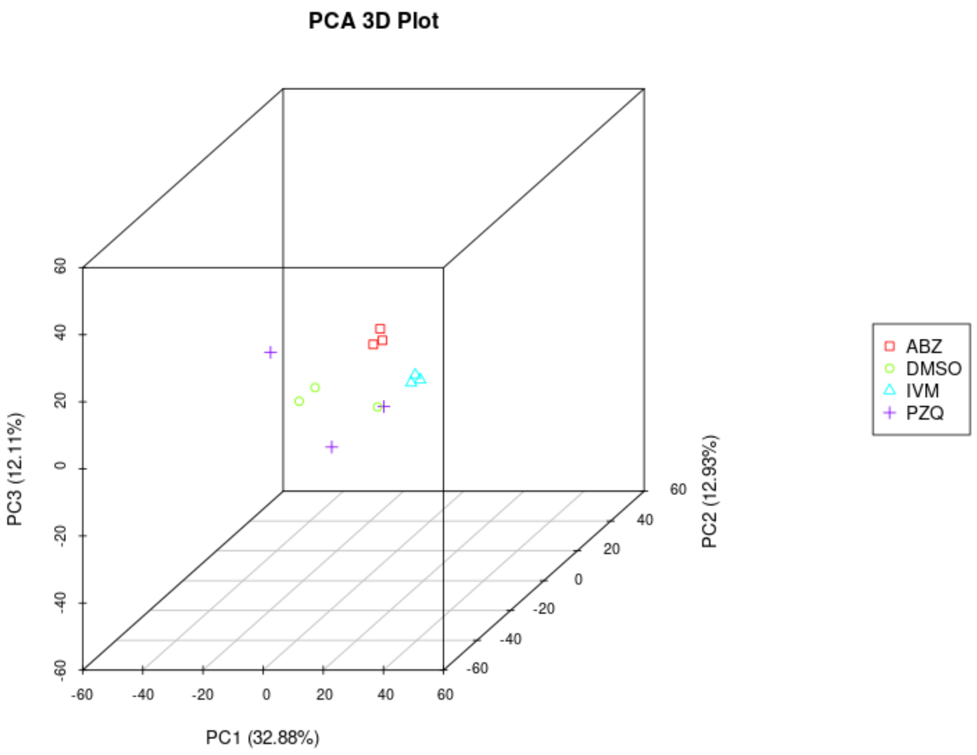

Figure S4. Principal component analysis (PCA) of gene expression profiles in drug-treated FHs 74 Int cells. PCA plot based on normalized gene expression values (FPKM) from all samples. Each point represents a sample, and the distances between points reflect similarities or differences in global gene expression profiles among experimental groups.
