# Supplementary material for: Effects of Three Anthelmintics on the Transcriptome of FHs 74 Int Enterocytes
Source: Trop Med Infect Dis. 2026 Sep 10;11(9):257. doi: 10.3390/tropicalmed11090257 (PMC13611716; doi:10.3390/tropicalmed11090257)
Supplement: Supplementary file 1 [file tropicalmed-11-00257-s001.zip › Table S4.pdf]

Table S4: The up/down-regulated DEGs at log_2 fold change ≥ 2, padj ≤ 0.05 following 1 μM albendazole treatment for 24 hours.

no	log2FC	padj	ID	description
				**UPREGULATED**
1	2.605	3.386E-22	POSTN	periostin [HGNC:16953]
2	2.365	3.024E-04	RN7SL2	RNA7SL cytoplasmic 2 [HGNC:23134]
3	5.897	2.274E-03	APOBEC3B	apolipoprotein B mRNA editing enzyme catalytic subunit 3B [HGNC:17352]
4	2.382	2.913E-03	SLC14A1	solute carrier family 14 member 1 (Kidd blood group) [HGNC:10918]
5	2.182	4.519E-03	RGPD5	RANBP2-like and GRIP domain containing 5 [HGNC:32418]
6	2.711	5.299E-03	KIF4A	kinesin family member 4A [HGNC:13339]
7	4.126	5.957E-03	SKA1	spindle and kinetochore associated complex subunit 1 [HGNC:28109]
8	2.266	6.634E-03	INMT	indolethylamine N-methyltransferase [HGNC:6069]
9	3.298	9.116E-03	IQGAP3	IQ motif containing GTPase activating protein 3 [HGNC:20669]
10	2.805	1.597E-02	PIMREG	PICALM interacting mitotic regulator [HGNC:25483]
11	3.134	2.030E-02	KIF18B	kinesin family member 18B [HGNC:27102]
12	4.240	2.106E-02	CENPM	centromere protein M [HGNC:18352]
13	2.997	2.171E-02	CENPA	centromere protein A [HGNC:1851]
14	3.168	2.516E-02	NEK2	NIMA related kinase 2 [HGNC:7745]
15	2.044	3.744E-02	ASF1B	anti-silencing function 1B histone chaperone [HGNC:20996]
16	2.010	4.490E-02	TMEM238	transmembrane protein 238 [HGNC:40042]
17	2.202	4.790E-02	BUB1	BUB1 mitotic checkpoint serine/threonine kinase [HGNC:1148]
				**DOWNREGULATED**
1	-2.715	1.676E-03	HIST2H4B	histone cluster 2 H4 family member b [HGNC:29607]
2	-2.024	1.415E-02	ATF3	activating transcription factor 3 [HGNC:785]

HGNC: HUGO Gene Nomenclature Committee
