# Supplementary material for: Effects of Three Anthelmintics on the Transcriptome of FHs 74 Int Enterocytes
Source: Trop Med Infect Dis. 2026 Sep 10;11(9):257. doi: 10.3390/tropicalmed11090257 (PMC13611716; doi:10.3390/tropicalmed11090257)
Supplement: Supplementary file 1 [file tropicalmed-11-00257-s001.zip › Table S5.pdf]

Table S5: The upregulated DEGs at log_2 fold change ≥ 2, padj ≤ 0.05 following 15 μM ivermectin treatment for 24 hours.

no	log2FC	padj	ID	description
1	2.284	1.363E-58	MTHFD2	methylenetetrahydrofolate dehydrogenase (NADP+ dependent) 2, methenyltetrahydrofolate cyclohydrolase [HGNC:7434]
2	3.259	3.511E-56	SLC7A1	solute carrier family 7 member 1 [HGNC:11057]
3	2.829	3.959E-49	PSAT1	phosphoserine aminotransferase 1 [HGNC:19129]
4	2.394	1.821E-45	PCK2	phosphoenolpyruvate carboxykinase 2, mitochondrial [HGNC:8725]
5	2.009	3.163E-38	NUPR1	nuclear protein 1, transcriptional regulator [HGNC:29990]
6	3.340	1.193E-37	PHGDH	phosphoglycerate dehydrogenase [HGNC:8923]
7	3.182	1.961E-34	CHAC1	ChaC glutathione specific gamma-glutamylcyclotransferase 1 [HGNC:28680]
8	2.618	1.156E-31	ASS1	argininosuccinate synthase 1 [HGNC:758]
9	2.673	1.491E-31	TRIB3	tribbles pseudokinase 3 [HGNC:16228]
10	2.797	5.068E-29	DHRS3	dehydrogenase/reductase 3 [HGNC:17693]
11	2.314	1.072E-28	EIF4EBP1	eukaryotic translation initiation factor 4E binding protein 1 [HGNC:3288]
12	2.119	9.725E-28	AGT	angiotensinogen [HGNC:333]
13	3.704	6.291E-27	SLC14A1	solute carrier family 14 member 1 (Kidd blood group) [HGNC:10918]
14	2.625	2.179E-24	TSPAN10	tetraspanin 10 [HGNC:29942]
15	2.437	4.021E-23	BCAT1	branched chain amino acid transaminase 1 [HGNC:976]
16	2.547	2.199E-20	PYCR1	pyrroline-5-carboxylate reductase 1 [HGNC:9721]
17	2.204	1.341E-17	SLC6A9	solute carrier family 6 member 9 [HGNC:11056]
18	2.042	3.021E-16	HMGA1	high mobility group AT-hook 1 [HGNC:5010]
19	3.017	3.238E-16	MAGED4	MAGE family member D4 [HGNC:23793]
20	2.058	2.374E-15	ATP6V0D2	ATPase H+ transporting V0 subunit d2 [HGNC:18266]
21	2.808	3.229E-14	CLDN1	claudin 1 [HGNC:2032]
22	2.251	7.883E-14	DLL4	delta like canonical Notch ligand 4 [HGNC:2910]
23	3.962	3.045E-12	STC2	stanniocalcin 2 [HGNC:11374]
24	4.367	3.206E-12	ADM2	adrenomedullin 2 [HGNC:28898]
25	2.302	1.481E-10	SCG2	secretogranin II [HGNC:10575]
26	3.330	9.315E-09	AC073283.2	novel transcript
27	2.578	2.788E-08	PTGER1	prostaglandin E receptor 1 [HGNC:9593]
28	2.582	6.845E-08	KCNN4	potassium calcium-activated channel subfamily N member 4 [HGNC:6293]
29	2.795	1.901E-07	KIF17	kinesin family member 17 [HGNC:19167]
30	2.156	3.047E-07	RASL10B	RAS like family 10 member B [HGNC:30295]
31	2.594	4.511E-07	PLPP2	phospholipid phosphatase 2 [HGNC:9230]
32	2.148	6.899E-07	LRRC32	leucine rich repeat containing 32 [HGNC:4161]
33	3.546	1.196E-06	JPH2	junctophilin 2 [HGNC:14202]
34	2.046	1.248E-06	PDGFB	platelet derived growth factor subunit B [HGNC:8800]
35	2.785	1.507E-06	ULBP1	UL16 binding protein 1 [HGNC:14893]
36	3.915	1.946E-06	ASNS	asparagine synthetase (glutamine-hydrolyzing) [HGNC:753]
37	2.088	2.561E-06	HSPB7	heat shock protein family B (small) member 7 [HGNC:5249]
38	2.900	2.785E-06	TNFRSF12A	TNF receptor superfamily member 12A [HGNC:18152]
39	2.385	7.806E-06	MMP1	matrix metallopeptidase 1 [HGNC:7155]
40	2.370	7.962E-06	DIRC3	disrupted in renal carcinoma 3 [HGNC:17805]
41	3.494	1.181E-05	MKX	mohawk homeobox [HGNC:23729]
42	4.032	2.152E-05	NDUFA4L2	NDUFA4, mitochondrial complex associated like 2 [HGNC:29836]
43	2.087	2.229E-05	LRRN4CL	LRRN4 C-terminal like [HGNC:33724]
44	2.426	2.450E-05	MRGPRF-AS1	MRGPRF antisense RNA 1 [HGNC:51140]

45	2.639	3.977E-05	SERPINE1	serpin family E member 1 [HGNC:8583]
46	2.893	4.007E-05	AC002401.4	novel transcript
47	2.448	4.722E-05	CNN1	calponin 1 [HGNC:2155]
48	3.246	5.622E-05	FABP3	fatty acid binding protein 3 [HGNC:3557]
49	2.077	6.403E-05	NEDD9	neural precursor cell expressed, developmentally down-regulated 9 [HGNC:7733]
50	2.706	8.149E-05	OXTR	oxytocin receptor [HGNC:8529]
51	2.427	1.080E-04	SH2D5	SH2 domain containing 5 [HGNC:28819]
52	2.414	1.397E-04	AC011511.5	novel transcript, antisense to ICAM4 and ICAM1
53	2.523	1.823E-04	NSFP1	N-ethylmaleimide-sensitive factor pseudogene 1 [HGNC:31405]
54	2.103	1.881E-04	INMT	indolethylamine N-methyltransferase [HGNC:6069]
55	2.510	3.498E-04	B4GALNT2	beta-1,4-N-acetyl-galactosaminyltransferase 2 [HGNC:24136]
56	3.887	3.524E-04	IL1RN	interleukin 1 receptor antagonist [HGNC:6000]
57	2.425	4.107E-04	IL20RB	interleukin 20 receptor subunit beta [HGNC:6004]
58	2.320	5.064E-04	FAM129A	family with sequence similarity 129 member A [HGNC:16784]
59	2.407	5.064E-04	RTN4R	reticulon 4 receptor [HGNC:18601]
60	2.703	5.441E-04	KRT80	keratin 80 [HGNC:27056]
61	3.402	6.112E-04	AL513329.1	novel transcript
62	2.445	6.272E-04	MYEOV	myeloma overexpressed [HGNC:7563]
63	3.014	6.319E-04	SCIN	scinderin [HGNC:21695]
64	2.723	7.282E-04	WTAPP1	Wilms tumor 1 associated protein pseudogene 1 [HGNC:44115]
65	2.980	8.555E-04	SIGLEC15	sialic acid binding Ig like lectin 15 [HGNC:27596]
66	2.046	1.395E-03	KLF15	Kruppel like factor 15 [HGNC:14536]
67	2.249	1.835E-03	TMEM238	transmembrane protein 238 [HGNC:40042]
68	2.303	2.116E-03	LINC02407	long intergenic non-protein coding RNA 2407 [HGNC:53336]
69	2.035	2.191E-03	NT5E	5'-nucleotidase ecto [HGNC:8021]
70	4.730	2.688E-03	Z94160.2	novel transcript, antisense to ELFN2
71	3.519	3.490E-03	KLHDC7B	kelch domain containing 7B [HGNC:25145]
72	2.136	4.050E-03	IL2RB	interleukin 2 receptor subunit beta [HGNC:6009]
73	2.371	4.939E-03	AMZ1	archaelysin family metallopeptidase 1 [HGNC:22231]
74	3.395	4.959E-03	KRTAP1-5	keratin associated protein 1-5 [HGNC:16777]
75	2.478	5.092E-03	AC087752.3	novel transcript
76	2.292	5.131E-03	GAS6-DT	GAS6 divergent transcript [HGNC:43694]
77	2.278	5.267E-03	TAGLN	transgelin [HGNC:11553]
78	2.091	5.448E-03	PRKCZ-AS1	PRKCZ antisense RNA 1 [HGNC:40477]
79	2.085	6.847E-03	ACTG2	actin, gamma 2, smooth muscle, enteric [HGNC:145]
80	3.615	7.494E-03	KCNE1B	potassium voltage-gated channel subfamily E regulatory subunit 1B [HGNC:52280]
81	2.165	8.048E-03	RCSD1	RCSD domain containing 1 [HGNC:28310]
82	2.456	1.012E-02	FOXN3-AS1	FOXN3 antisense RNA 1 [HGNC:44290]
83	2.144	1.078E-02	CBS	cystathionine-beta-synthase [HGNC:1550]
84	2.273	1.193E-02	AC006030.1	artifact
85	2.344	1.231E-02	AC105105.1	novel transcript, antisense to ALPK2
86	2.266	1.235E-02	ZP3	zona pellucida glycoprotein 3 [HGNC:13189]
87	2.058	1.376E-02	LINC01836	long intergenic non-protein coding RNA 1836 [HGNC:52652]
88	2.141	1.474E-02	HOPX	HOP homeobox [HGNC:24961]
89	2.469	1.678E-02	FLNC-AS1	FLNC antisense RNA 1 [HGNC:53474]
90	2.211	1.680E-02	FER1L6	fer-1 like family member 6 [HGNC:28065]
91	2.207	1.702E-02	ZBED3-AS1	ZBED3 antisense RNA 1 [HGNC:44188]
92	2.300	1.772E-02	SLC37A2	solute carrier family 37 member 2 [HGNC:20644]
93	2.762	1.849E-02	AC016757.1	uncharacterized LOC151174 [Source:NCBI gene;Acc:151174]
94	2.479	1.856E-02	INHBB	inhibin subunit beta B [HGNC:6067]
95	2.421	2.005E-02	KIRREL3	kirre like nephrin family adhesion molecule 3 [HGNC:23204]

96	2.200	2.246E-02	NXPH4	neurexophilin 4 [HGNC:8078]
97	2.671	2.309E-02	SOWAHD	sosondowah ankyrin repeat domain family member D [HGNC:32960]
98	2.529	2.587E-02	BGN	biglycan [HGNC:1044]
99	2.517	2.588E-02	MESP1	mesoderm posterior bHLH transcription factor 1 [HGNC:29658]
100	2.109	2.881E-02	AC007378.1	novel transcript
101	2.243	3.601E-02	NMRAL2P	NmrA like redox sensor 2, pseudogene [HGNC:52332]
102	2.574	3.746E-02	AC145207.4	novel transcript, antisense PYCR1
103	2.178	4.301E-02	BAIAP2L2	BAI1 associated protein 2 like 2 [HGNC:26203]
104	2.181	4.475E-02	DUSP9	dual specificity phosphatase 9 [HGNC:3076]
105	2.409	4.758E-02	CEACAMP10	carcinoembryonic antigen related cell adhesion molecule pseudogene 10 [HGNC:1822]
106	2.043	4.784E-02	AC137932.3	novel transcript, antisense to ANKRD11

HGNC: HUGO Gene Nomenclature Committee
