# Supplementary material for: Effects of Three Anthelmintics on the Transcriptome of FHs 74 Int Enterocytes
Source: Trop Med Infect Dis. 2026 Sep 10;11(9):257. doi: 10.3390/tropicalmed11090257 (PMC13611716; doi:10.3390/tropicalmed11090257)
Supplement: Supplementary file 1 [file tropicalmed-11-00257-s001.zip › Table S6.pdf]

Table S6: The downregulated DEGs at log_2 fold change ≥ 2, padj ≤ 0.05 following 15 μM ivermectin treatment for 24 hours.

no	log2FC	padj	ID	description
1	-5.296	5.953E-74	FOSB	FosB proto-oncogene, AP-1 transcription factor subunit [HGNC:3797]
2	-2.875	2.440E-61	PDK4	pyruvate dehydrogenase kinase 4 [HGNC:8812]
3	-3.181	1.314E-49	NR4A1	nuclear receptor subfamily 4 group A member 1 [HGNC:7980]
4	-2.353	4.892E-28	ZFP36	ZFP36 ring finger protein [HGNC:12862]
5	-3.540	2.508E-27	DUSP2	dual specificity phosphatase 2 [HGNC:3068]
6	-2.183	2.532E-21	TRPC3	transient receptor potential cation channel subfamily C member 3 [HGNC:12335]
7	-2.093	7.501E-19	AL391056.1	novel transcript (FLJ20378)
8	-2.242	7.478E-18	NR4A2	nuclear receptor subfamily 4 group A member 2 [HGNC:7981]
9	-2.400	1.230E-16	SSTR2	somatostatin receptor 2 [HGNC:11331]
10	-2.977	1.242E-15	NPY1R	neuropeptide Y receptor Y1 [HGNC:7956]
11	-2.028	3.588E-14	TACR1	tachykinin receptor 1 [HGNC:11526]
12	-2.259	1.483E-13	AC093908.1	TEC
13	-2.138	2.384E-13	TXNDC16	thioredoxin domain containing 16 [HGNC:19965]
14	-2.151	3.615E-13	FAM19A2	family with sequence similarity 19 member A2, C-C motif chemokine like [HGNC:21589]
15	-2.623	1.375E-12	ANGPTL4	angiopoietin like 4 [HGNC:16039]
16	-3.009	1.607E-10	SLC6A4	solute carrier family 6 member 4 [HGNC:11050]
17	-2.325	2.470E-10	AL391427.1	uncharacterized LOC101928051 [NCBI gene:101928051]
18	-3.052	2.796E-10	MYOCD	myocardin [HGNC:16067]
19	-2.965	4.970E-10	PDE10A	phosphodiesterase 10A [HGNC:8772]
20	-2.324	1.108E-09	SPON1	spondin 1 [HGNC:11252]
21	-2.003	4.798E-09	CXCL2	C-X-C motif chemokine ligand 2 [HGNC:4603]
22	-2.069	1.491E-08	ALKAL2	ALK and LTK ligand 2 [HGNC:27683]
23	-2.033	1.634E-08	PRRG4	proline rich and Gla domain 4 [HGNC:30799]
24	-3.882	1.698E-07	FOS	Fos proto-oncogene, AP-1 transcription factor subunit [HGNC:3796]
25	-2.603	1.048E-06	ID4	inhibitor of DNA binding 4, HLH protein [HGNC:5363]
26	-2.038	3.145E-05	EDNRA	endothelin receptor type A [HGNC:3179]
27	-2.616	3.309E-05	AC025259.3	novel transcript
28	-2.288	4.538E-05	SEMA3D	semaphorin 3D [HGNC:10726]
29	-2.942	5.218E-05	ENPP2	ectonucleotide pyrophosphatase/phosphodiesterase 2 [HGNC:3357]
30	-3.734	6.403E-05	WNT2	Wnt family member 2 [HGNC:12780]
31	-3.952	7.157E-05	SCARA5	scavenger receptor class A member 5 [HGNC:28701]
32	-2.113	9.997E-05	EGR1	early growth response 1 [HGNC:3238]
33	-3.108	1.481E-04	EGR2	early growth response 2 [HGNC:3239]
34	-3.888	1.591E-04	AL137058.1	transmembrane phosphatase with tensin homology (TPTE) pseudogene
35	-2.184	2.510E-04	AC243772.2	novel transcript, antisense to FCGR1A
36	-2.199	2.645E-04	KIF5C	kinesin family member 5C [HGNC:6325]
37	-2.297	6.131E-04	DLX3	distal-less homeobox 3 [HGNC:2916]
38	-2.146	6.272E-04	EGR3	early growth response 3 [HGNC:3240]
39	-2.976	7.078E-04	CFTR	cystic fibrosis transmembrane conductance regulator [HGNC:1884]
40	-2.410	1.773E-03	HTR2A	5-hydroxytryptamine receptor 2A [HGNC:5293]
41	-4.137	1.786E-03	TENM2	teneurin transmembrane protein 2 [HGNC:29943]
42	-3.297	2.212E-03	SIK1	salt inducible kinase 1 [HGNC:11142]
43	-2.318	2.408E-03	NR4A3	nuclear receptor subfamily 4 group A member 3 [HGNC:7982]
44	-2.258	2.673E-03	PTGS2	prostaglandin-endoperoxide synthase 2 [HGNC:9605]
45	-2.826	2.847E-03	PPEF1	protein phosphatase with EF-hand domain 1 [HGNC:9243]

46	-2.078	3.586E-03	TCIM	transcriptional and immune response regulator [HGNC:1357]
47	-2.474	3.753E-03	HPCAL4	hippocalcin like 4 [HGNC:18212]
48	-2.753	4.212E-03	KIF20A	kinesin family member 20A [HGNC:9787]
49	-2.279	5.472E-03	ADGRL2	adhesion G protein-coupled receptor L2 [HGNC:18582]
50	-2.422	5.472E-03	AC004846.1	uncharacterized LOC101928123 [NCBI gene:101928123]
51	-2.377	6.379E-03	RNF150	ring finger protein 150 [HGNC:23138]
52	-2.029	6.775E-03	DCLK1	doublecortin like kinase 1 [HGNC:2700]
53	-2.556	6.943E-03	ZNF711	zinc finger protein 711 [HGNC:13128]
54	-3.025	7.425E-03	CTD-2201I18.1	uncharacterized LOC101929215 [NCBI gene:101929215]
55	-2.060	1.117E-02	MAOA	monoamine oxidase A [HGNC:6833]
56	-2.615	1.909E-02	COL23A1	collagen type XXIII alpha 1 chain [HGNC:22990]
57	-2.771	2.659E-02	EMILIN3	elastin microfibril interfacer 3 [HGNC:16123]
58	-2.387	2.958E-02	ZCCHC12	zinc finger CCHC-type containing 12 [HGNC:27273]
59	-2.838	2.982E-02	MIR181A1HG	MIR181A1 host gene [HGNC:48659]
60	-2.537	3.013E-02	PGM5	phosphoglucomutase 5 [HGNC:8908]
61	-2.412	3.297E-02	IL1R2	interleukin 1 receptor type 2 [HGNC:5994]
62	-2.111	3.539E-02	LINC01152	long intergenic non-protein coding RNA 1152 [HGNC:16752]
63	-2.505	4.253E-02	AC091814.1	novel transcript
64	-2.946	4.469E-02	PWP2	PWP2, small subunit processome component [HGNC:9711]
65	-2.316	4.771E-02	KIF14	kinesin family member 14 [HGNC:19181]

HGNC: HUGO Gene Nomenclature Committee
